# Revisiting the impact of *Schistosoma mansoni* regulating mechanisms on transmission dynamics using SchiSTOP, a novel modelling framework

**DOI:** 10.1371/journal.pntd.0012464

**Published:** 2024-09-20

**Authors:** Veronica Malizia, Sake J. de Vlas, Kit C. B. Roes, Federica Giardina

**Affiliations:** 1 Department of Public Health, Erasmus MC, University Medical Center Rotterdam, Rotterdam, The Netherlands; 2 Radboud University Medical Center, Department IQ Health, Biostatistics Research Group, Nijmegen, The Netherlands; Oregon State University College of Veterinary Medicine, UNITED STATES OF AMERICA

## Abstract

**Background:**

The transmission cycle of *Schistosoma* is remarkably complex, including sexual reproduction in human hosts and asexual reproduction in the intermediate host (freshwater snails). Patterns of rapid recrudescence after treatment and stable low transmission are often observed, hampering the achievement of control targets. Current mathematical models commonly assume regulation of transmission to occur at worm level through density-dependent egg production. However, conclusive evidence on this regulating mechanism is weak, especially for *S*. *mansoni*. In this study, we explore the interplay of different regulating mechanisms and their ability to explain observed patterns in *S*. *mansoni* epidemiology.

**Methodology/Principal findings:**

We developed SchiSTOP: a hybrid stochastic agent-based and deterministic modelling framework for *S*. *mansoni* transmission in an age-structured human population. We implemented different models with regulating mechanisms at: i) worm-level (density-dependent egg production), ii) human-level (anti-reinfection immunity), and iii) snail-level (density-dependent snail dynamics). Additionally, we considered two functional choices for the age-specific relative exposure to infection. We assessed the ability of each model to reproduce observed epidemiological patterns pre- and post-control, and compared successful models in their predictions of the impact of school-based and community-wide treatment.

Simulations confirmed that assuming at least one regulating mechanism is required to reproduce a stable endemic equilibrium. Snail-level regulation was necessary to explain stable low transmission, while models combining snail- and human-level regulation with an age-exposure function informed with water contact data were successful in reproducing a rapid rebound after treatment. However, the predicted probability of reaching the control targets varied largely across models.

**Conclusions/Significance:**

The choice of regulating mechanisms in schistosomiasis modelling largely determines the expected impact of control interventions. Overall, this work suggests that reaching the control targets solely through mass drug administration may be more challenging than currently thought. We highlight the importance of regulating mechanisms to be included in transmission models used for policy.

## Author summary

Schistosomiasis is a neglected tropical disease estimated to affect 250 million people worldwide and causing severe morbidity in endemic countries. The transmission of *Schistosoma* is complicated by the multiplication of parasites within freshwater snails that act as obligate intermediate hosts. Rapid rebound of infection after mass treatment is commonly observed, as well as stable low transmission. Several mathematical models are currently employed to inform policy decisions, building on the predominant assumption of worm-level regulation through density-dependent egg production. We explored the interplay of different regulating mechanisms assumed at worm-, human-, and snail-level and their ability to explain observed epidemiological patterns. To this end, we developed SchiSTOP, a new modelling framework for *S*. *mansoni* transmission dynamics and considered 162 model variants. Results show that snail-level regulation is required to explain stable low transmission, while the combination of snail- and human-level regulation is necessary to reproduce a rapid rebound after control. We further show that assumptions on the regulating mechanisms heavily influence conclusions about the feasibility to reach control targets. In particular, the models that successfully explain observed patterns for *S*. *mansoni* suggest that achieving control targets solely through mass drug administration may be more challenging than currently thought.

## Introduction

Schistosomiasis is a neglected tropical disease (NTD) caused by parasitic flatworm (*Schistosoma)* species. Widespread in tropical and sub-tropical regions, schistosomiasis is endemic in 78 countries with an estimated 251.4 million people requiring treatment in 2021 [1]. Responsible for severe morbidity such as complications due to chronic infections of the urinary tract or the intestine, schistosomiasis is considered the deadliest among NTDs [2]. The intestinal form of schistosomiasis is caused by *S*. *mansoni*, and the urogenital one is caused by *S*. *haematobium*, two of the main species responsible of human infections [3]. The transmission dynamics of *Schistosoma* between the human host and the water environment are peculiar and remarkably complex, due to the presence of two reproduction phases. The first-stage larvae (free-living miracidia) are released from eggs hatching upon contact with freshwater, which search for certain species of freshwater snails. Asexual reproduction occurs in the snails, leading to the release of second-stage larvae (free-living cercariae). These cercariae can infect humans, where they mature into adult worms, pair and reproduce sexually. Monogamous in their nature, adult pairs of *Schistosoma* release eggs within the human host. Individuals with schistosomiasis contaminate freshwater sources with urine (*S*. *haematobium*) or faeces (*S*. *mansoni*) containing parasite eggs. The World Health Organization (WHO) 2030 Roadmap for NTDs delineates two control targets for schistosomiasis: elimination as a public health problem (EPHP) in all endemic countries and interruption of transmission (IOT) in 32% of the endemic countries [1]. EPHP is defined by WHO as achieving a prevalence of heavy intensity infections in school-aged children (SAC) below 1%. Infections are classified as heavy intensity if more than 400 eggs per gram of faeces (epg) are detected for *S*. *mansoni*, and more than 50 eggs per 10 mL urine for *S*. *haematobium*. IOT is defined by WHO as having no new autochthonous human cases in a defined geographical area.

Mass drug administration (MDA) to SAC with the anthelmintic drug praziquantel is the primary measure for the control of schistosomiasis. Praziquantel reduces the overall prevalence of infection in the population by killing adult worms within the human hosts [4]. However, the prevalence of infection typically rebounds to pre-control levels quickly after stopping MDA [1,5,6], but we currently lack a clear understanding of rebound pathways. Moreover, maintained transmission at low endemic levels (<10% prevalence in SAC) has been observed [7–9], hampering the achievement of IOT. The WHO has recently provided a recommendation for additional control measures to prevent rebound of infection, including focal snail control and behavioural change interventions (through education and improvement of hygiene and sanitation standards) in endemic countries [10].

Mathematical models have historically been employed to reproduce and elucidate the transmission dynamics of *Schistosoma* species [11]. Deterministic models have been developed to describe the mean worm burden within humans over time [12–15], while stochastic agent-based models (ABMs) have included individual heterogeneities in transmission, exposure to infection, or adherence to treatment [16–21]. Mathematical models for schistosomiasis require assumptions on mechanisms regulating transmission in order to reproduce a stable endemic equilibrium. In the absence of regulating mechanisms, the number of cercariae in the environment and the burden of worms in humans will grow unbounded, leading to an endemicity setting where the entire population is heavily infected. The assumption of density-dependence in egg production, according to which production of eggs by female worms diminishes with increasing adult worm burdens (density-dependent fecundity), has been largely accepted and employed with the rationale of overcrowding effects within the human host, in analogy with other intestinal parasites [13,22]. However, scientific evidence for this assumption for schistosomiasis has been based on historic human autopsy data [23,24], and the applicability of such data was disputed [25]. The availability of new molecular data analysed via sibship reconstruction [26] has provided some recent evidence of density-dependence in egg production for *S*. *haematobium* species, but the results were not conclusive for *S*. *mansoni*, where this question remains unresolved.

Regulating mechanisms at other stages of the transmission cycle occur, however the interplay of such mechanisms has not been generally included in current models used for policy. At the human level, host-acquired immunity has been suggested in antibodies detection studies [27–29] and was included in some deterministic age-structured models [30–32]. However, a clear and conclusive understanding of the processes of acquired immunity (e.g. triggers, rate of development) and the extent to which it may restrain transmission is missing, especially for *S*. *mansoni* [33]. In addition, modelling studies have shed some light on the role of the intermediate host in the transmission dynamics of schistosomiasis [34–36]. Snails can regulate transmission dynamics in two ways. Firstly, they can amplify transmission due to the parasite asexual reproduction phase occurring within the snail host. Conversely, they can also limit transmission because of factors such as the depletion of susceptible snails, castration, and increased snail mortality upon infection [37,38]. These factors on their turn are influenced by the overall abundance of snails, which is determined by intraspecific competition for resources [36]. Overall, insufficient attention has been given to describe both host-acquired immunity and the explicit dynamics of the intermediate host in mathematical models employed for guiding policy decisions. There is a need to further understand the extent to which different regulating mechanisms occurring at worm-, human-, and snail-level can explain observed epidemiological patterns and how these affect the estimated impact of control interventions.

The aim of this study is to assess how well different modelling assumptions on the regulating mechanisms of schistosomiasis transmission dynamics can reproduce observed endemicity settings (low, moderate, high) and explain epidemiological patterns observed before, during, and after MDA. We also investigate how such assumptions on regulating mechanisms and their interplay affect the feasibility to reach the control targets set by the WHO, in particular EPHP and IOT, following two MDA strategies: targeting SAC or the whole community. In this work, we focused on *S*. *mansoni* species and developed SchiSTOP: a hybrid modelling framework for the transmission of *S*. *mansoni* between humans and snails that combines an agent-based model (ABM) for the human and worm populations, and a deterministic compartmental model for the snail dynamics. We consider the combinations of three assumptions on the regulation occurring at worm-level (density-dependence in egg production), at human-level (anti-reinfection immunity), and at snail-level (density-dependence in snails’ population growth).

## Methods

### Model structure

We developed SchiSTOP, an agent-based stochastic modelling framework for the transmission dynamics of schistosomiasis between the human hosts and the contaminated water environment via larvae multiplication in the intermediate host (freshwater snails). The dynamics of the snail population are included as an explicit deterministic compartmental model integrated into the ABM.

SchiSTOP consists of five main building blocks: the human population, the parasitic worms living in the human host, the two larval stages living freely in the contaminated water environment, and the snail population. The models include human, worm, and snail population dynamics via births, aging, and deaths. We focus this section on the definition of the different regulating mechanisms and the simulation scheme. However, thorough specifications of SchiSTOP, including human demography, human and snail infection dynamics, diagnostic procedure, and mass drug administration, are provided in **S1 Text**.

**Fig 1:**
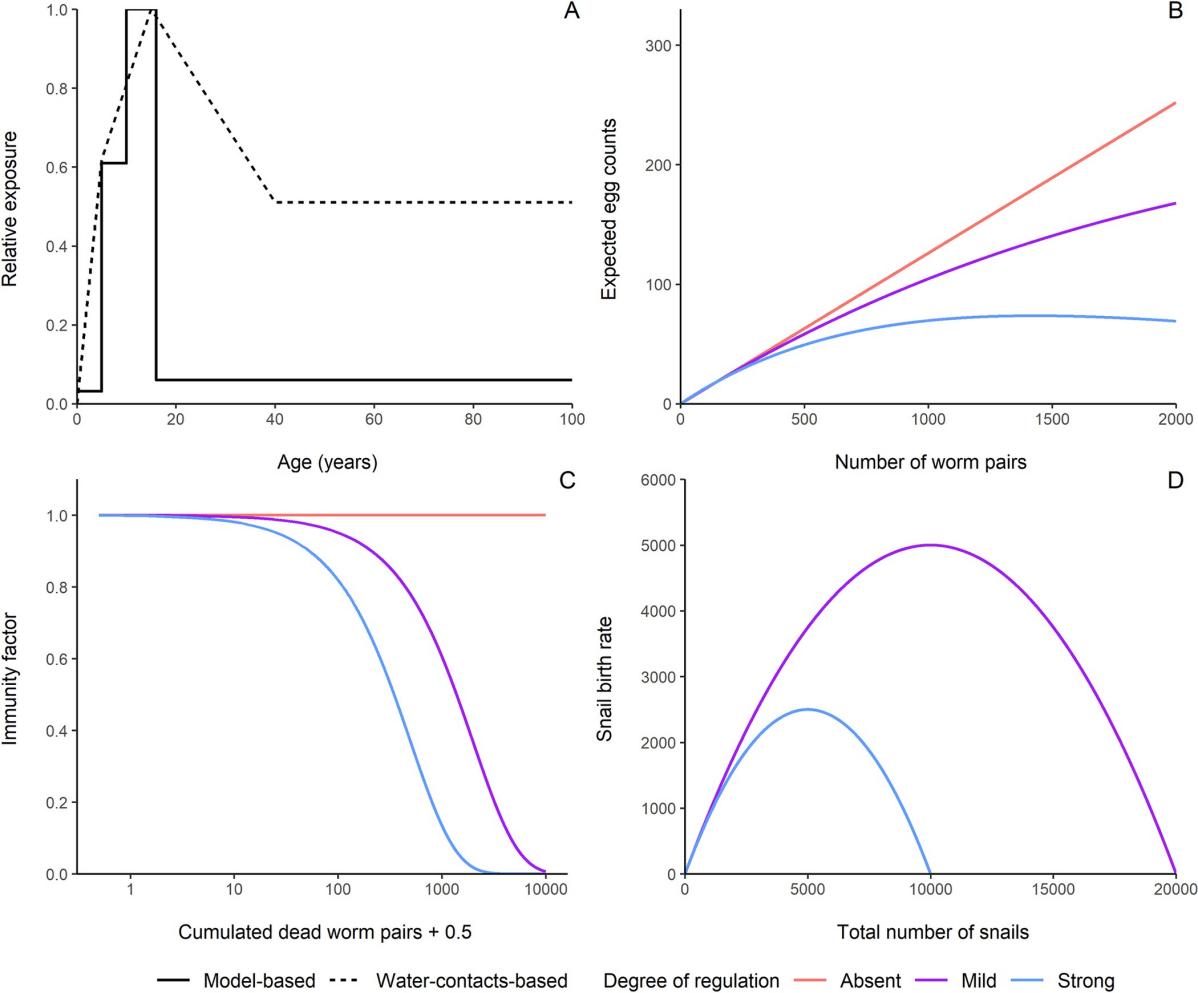
Modelling assumptions. **(A) Age-exposure functions employed in the models.** The functions describe the relative exposure by age of the human host, using 1.0 for the highest value (i.e., at age 15). This is proportional to the individual overall exposure rate. Both functions are interpolations of reported data points: the solid line is a piece-wise constant function representing the relative exposures derived from literature (model-based exposure function) at 0, 5, 10, and 16+ years of age [21]. The dashed line is a piece-wise linear function based on the relative exposures extracted from published water contacts data at 0, 5, 15, and 40+ years of age [41,42]. **(B) Worm-level regulation: density-dependence in egg production.** The expected egg counts are function of the number of mature worms, which are modelled as pairs within the human host. The mean number of eggs per pair per sample *α* in this figure is set at 0.13 (Absent regulation), 0.13 (Mild regulation), or 0.14 (Strong regulation). The density-dependence parameter z is set at 0 (Absent regulation), 0.00022 (Mild regulation), or 0.0007 (Strong regulation). See text for the parameterization of *α* and z for different degree of worm-level regulation. **(C) Human-level regulation: (dying-worm driven) anti-reinfection immunity.** The immunity factor is displayed as a function of accumulated dead worms, presented on a logarithmic scale. The immunity coefficient *α_imm_* is 0 (Absent), 0.0005 (Mild), or 0.002 (Strong). **(D) Snail-level regulation: density-dependent population growth.** The snail birth rate, following a logistic growth, is displayed as a function of the total number of snails. The snail carrying capacity K is set at 20000 (Mild), or 10000 (Strong). When no regulation at the snail-level is assumed, the model does not include explicit snail dynamics, therefore the corresponding degree is not displayed.

The ABM is based on stochastic events updated at discrete time steps *t* of one month. We assume that the model replicates the transmission dynamics in a rural community, simulating a human population of 1000 individuals, structured by age. The demographic dynamics are governed by a fixed birth rate and an age-specific death probability, informed by the Ugandan Bureau of Statistics [39]. Individuals can also migrate out of the population, according to a rate that results in a stable population size. The population is updated at each time step. Before starting simulations from the transmission model, we simulate the demographic dynamics until reaching an equilibrium for 200 years. The equilibrium age distribution (**Fig 1** in **S1 Text**) is then used to initialize the transmission model in successive iterations. Infection in humans is then modelled through an age-specific exposure to infection, accounting for individual patterns of contacts with water potentially contaminated with cercariae. Exposure to infection is governed by the overall transmission parameter on humans *ζ* and the level of worm aggregation *k*_*w*_. The distribution of worms in the human population is in fact highly aggregated, with most of the population harboring few or no parasites, and a few individuals harboring high worm burden. Maturation, reproduction, and death of worms within the human host are governed by stochastic events. Human hosts contribute to contaminate the water environment through excretion of eggs which hatch and release miracidia, which can infect snails. Infection dynamics in snails is explicitly modelled until the release of cercariae. Diagnostic schemes to assess infection in humans via the Kato-Katz technique, and control interventions via 10 annual rounds of MDA are explicitly modelled, assuming 75% MDA coverage of the target population, selected randomly [10]. This includes a 5% of the targeted population who systematically does not have access to treatment. We assume an imperfect drug efficacy of 86% of adult worms killed by MDA [40]. The full list of models’ parameters employed for the simulations of this study can be found in **S1 Table**.

SchiSTOP allows the implementation of different model variants (from here on called models) by varying assumptions on parameters and processes describing the dynamics of the disease. For the scope of this study, we considered and implemented 162 different models that reflect the 3^3^ = 27 combinations of three regulating mechanisms acting at different levels of the transmission cycle (worm, human, and snail) at three intensities (absent, mild, strong), for two different functions to model the age-specific exposure to infection, and three endemicity levels (low, moderate, high). In particular, the three regulating mechanisms act at: i) the worm-level, through density-dependence in egg production, ii) the human-level, through anti-reinfection immunity driven by dying worms, and iii) the snail-level, through the density-dependent growth of snail population. For simplicity, we refer to these three regulating mechanisms as worm-, human-, and snail-level regulation throughout the text. The two functional choices for the relative age-specific exposure are i) a model-based function derived from fitting an established schistosomiasis transmission model [21] to observed age-intensity profiles [40] (model-based), and ii) an alternative age-exposure function that we estimated from published water contact data [41,42] (water-contacts-based). To inform the latter, we extracted frequency of water contacts adjusted for body surface and time of day of contact reported for three age groups (0–9, 10–19, 20+ years old) from an extensive data collection on direct water contact observations in a village in Northern Senegal [41]. To refine the modelling of the exposure in the last age-group (20+), various age-exposure profiles from Kenya [42] were used. Both functions are implemented as relative exposure and displayed in **Fig 1A.**

#### Worm-level regulation: Density-dependent egg production

We consider mature worms to live in pairs within the human host. For each human individual *i*, the relationship between the expected excreted eggs (*μ_i_*) and the number of carried adult worm pairs (*w_i_*), is implemented as an exponential saturating function, describing density-dependent fecundity of female worms as follows: $\mu_{i}=\alpha w_{i}e^{-zw_{i}}$. In this function, the parameter α represents the maximum fecundity of female worms (i.e., the expected eggs produced per worm pair in absence of density-dependence), *z* is a parameter dictating the level of saturation in egg production, and therefore the strength of the density-dependent mechanism. The worm-level regulating mechanism for different values of the parameters α and *z* is displayed in **Fig 1B**. The values of α and *z* were set to numbers available from previous work or tuned to produce a mild or strong regulation. To do so, we first set *z* and α from literature [17,40,43] to parametrise a strong worm-level regulation. Secondly, we set *z* to 0 in absence of worm-level regulation, assuming the expected excreted eggs to linearly depend on the number of adult worm pairs and then tuned α to reproduce the same initial growth as in presence of strong density-dependence. Finally, we tuned both parameters for a mild worm-level regulation.

#### Human-level regulation: (dying-worm driven) anti-reinfection immunity

The human immunological response, which develops naturally, is assumed to be induced by the death of adult worms, as proposed in published immunological studies [33,44]. Anti-reinfection immunity protects the host from reinfection by hindering the maturation of cercariae into adult worms, and the amount of this immunity increases as a function of the cumulative number of died adult worms. The model describes this behaviour via an immunity factor given by $e^{-\alpha_{imm}d}$ ranging between 0 and 1, which reduces the rate of parasite acquisition by the human host. Here, *α_imm_* is the immunity parameter and *d* is the individual accumulated dead worm burden, defined in terms of worm pairs and inclusive of worms that have undergone either natural mortality or mortality due to MDA. We do not consider decay of immunity within the human lifespan. The human-level regulating mechanism for different values of *α_imm_* is displayed in **Fig 1C**. The immunity factor is multiplied times the exposure rate, which determines the force of infection acting on humans (*FOI*_*h*_). In absence of anti-reinfection immunity, we set *α_imm_* to 0, implying that the past exposure to infection (and therefore the death of worms) does not affect the chance to acquire new parasites. As a reference for a strong human-level regulation, we derived *α_imm_* from literature [32]. We set the parameter to a lower value to define a mild degree of regulation.

#### Snail-level regulation: density-dependent population growth

The snail phase of the parasite life cycle is explicitly modelled through an ODE-based framework and embedded into the ABM. We employed a deterministic compartmental model to simulate births, deaths, exposure, and infection of snails. Susceptible snails *S(t)* reproduce according to a logistic growth with birth rate *β(t)* due to competition for resources:

$$\beta (t)=\beta_{0}\left(1-\frac{N_{s}(t)}{K}\right)(S(t)+E(t))$$

Here, *β*_*0*_ is the maximum reproduction rate in absence of competition [36], *N*_*s*_*(t)* the total snail population size, and *K* the carrying capacity. The birth rate of snails, dictating the degree of the snail-level regulating mechanism, is displayed in **Fig 1D** for different values of *K*. The carrying capacity *K* was tuned to parametrise a mild or strong degree of snail-level regulation.

Susceptible snails can be invaded by miracidia present in the water environment after egg excretion by human hosts, and become exposed (*E(t)*), i.e., infected but not infectious. The force of infection *FOI*_*s*_ acting on snails is proportional to the abundance of miracidia, through a coefficient *η* representing the transmission parameter on snails. Miracidia live in exposed snails for a period during which they mature and asexually reproduce into cercariae. Susceptible and exposed snails undergo natural mortality, according to a per-capita mortality rate *ν* [36]. When the parasites are mature, the snails move to the infected compartment *I(t)* at a rate *τ* equal to the inverse of the assumed parasites’ maturation period [17,45]. Infection on snails causes an increased mortality (*ν*_*I*_) and castration [36]. Therefore, only susceptible and exposed snails contribute to reproduction (see formulation of *β(t)*). The depletion of susceptible snails and the effects of infection on the snail population growth, regulate the transmission of schistosomiasis at this stage of the transmission cycle. Infected snails shed cercariae *C(t)* into the environment, according to a cercarial per capita production rate *λ* [36]. The abundance of shed cercariae determines the force of infection acting on humans *FOI*_*h*_. Cercariae naturally die with a mortality rate *γ* before infecting humans. The dynamics of this module are thus described by the following system of ordinary differential equations.

$$\frac{dS(t)}{dt}=\beta -(v+FOI_{s})S(t)$$

$$\frac{dE(t)}{dt}=FOI_{s}S(t)-(v+\tau )E(t)$$

$$\frac{dI(t)}{dt}=\tau E(t)-v_{I}I(t)$$

$$\frac{dC(t)}{dt}=\lambda I(t)-\gamma C(t)$$

The system of ODEs is defined in continuous time with events governed by daily rates. At each time step of the agent-based model (discrete and set to one month), we simulate from the system of ODEs over a 1-month time horizon. The results from these simulations then serve as starting values for the following ABM time step (details in **S1 Text**).

In the scenario where no regulation at the snail-level is considered, the infection dynamics in the snail population are not explicitly modelled. We assume instead that the cercarial uptake by the environment at time *t* is equal to the number of miracidia shed at the previous time step, which coincides with the maturation period into the intermediate host (1 month). The full list of parameters can be found in **S1 Table.** The specifications of the snails’ infection dynamics can be found in **S1 Text, Intermediate host**.

### Simulations

Three parameters, namely the overall transmission parameter on humans *ζ*, the level of worm aggregation *k*_*w*_, and the transmission parameter on snails η, were varied within a grid search framework with the aim of allowing all models to reproduce the same initial setting of either low, moderate, or high endemicity. The initial settings were defined on the basis of three prevalence indicators and their targets informed from literature: the infection prevalence in SAC, as per WHO definition (average of 10%, 30%, 60%, in low, moderate, and high endemicity, respectively) [10], the prevalence of heavy infections in SAC (average of 0%, 6%, 20%) [46], and the prevalence of infection in snails (average of 6% in all settings) [37]. First, a visual inspection of the three prevalence indicators allowed refining the search. The prevalence of infection in snails is mostly influenced by the parameter η, about which very little information is available. We therefore varied the parameter η until the models reproduced a prevalence in snails in the range 6% ± 1%. Afterwards, we performed the grid search for the three transmission parameters jointly in the reduced space and identified for each model the parameters’ values minimising the root mean squared error (RMSE) between the predicted and desired targets. The RMSE was computed combining and scaling the three target indicators with most of the weight assigned to the prevalence of any infection in SAC, which was allowed to vary in relative terms about ± 10% with respect to the corresponding desired threshold (10 ± 1%, 30 ± 3%, 60 ± 6%, in low, moderate, and high endemicity, respectively). The file with selected parameters for each model and the full code is publicly available at https://github.com/VeronicaMalizia/SchiSTOP_model. SchiSTOP was implemented and run using R programming language 4.2.2 [47].

After the selection of parameters, we ran 100 stochastic simulations for each model to capture inherent models’ variability and used the average trends for comparison. We assessed and compared the ability of each model to explain three observable patterns: i) the age-intensity profiles expressed as mean detected eggs per gram (epg) of faeces as a function of age, ii) the effect of 10 annual rounds of MDA administered to SAC, iii) the rebound of prevalence after treatment. The number of eggs per gram of faeces (epg) is expressed as geometric mean across age groups (see **S1 Text, Human Demography**) and stochastic realizations of the models. Although geometric means lead to biased estimators of the true mean, they have traditionally been used in the analysis of data from field studies [48]. We defined models as successful when they could i) reproduce a convex age-intensity curve with a typical peak of intensity of infection in adolescents, ii) bring the prevalence of infection in the human population down of at least 20% of their pre-control levels after 10 years of MDA administered to SAC, and iii) result in a rebound of prevalence to values close to pre-control levels, after stopping MDA (10 annual rounds to SAC), even in low endemicity settings [1,49–51]. After stopping MDA, we expect that the prevalence of infection in SAC should go back to pre-control levels within 20 years. In order to parametrise the model and define the above-mentioned criteria for model plausibility, we undertook an expert elicitation process that involved multiple steps. We first identified experts in the field of schistosomiasis, ranging from parasitology, epidemiology, experimental and field research. All experts had a strong publication record, practical experience, and a deep understanding of the disease dynamics. All experts were informed about the purpose of the elicitation process, the goals of our research, and the specific areas in which we sought expertise. The experts involved are acknowledged at the end of this paper. In particular, we conducted structured interviews and one workshop, all organized within the SchiSTOP project [52]. We asked questions related to 1) the role of the intermediate host and how to interpret malacological surveys, 2) exposure and mechanisms of immunity in terms of triggers, protection, and decay and 3) prevalence patterns before and after control.

Lastly, we used the successful models to compare the predicted probabilities (based on 100 repeated simulation runs) to meet the control targets of EPHP and IOT, considering two different treatment strategies: 10 rounds of annual MDA administered to SAC (school-based), and 10 rounds of annual MDA administered to all individuals older than 2 (community-wide), as per the most recent WHO guidelines towards the 2030 targets [10]. EPHP is reached when the prevalence of heavy intensity of infections in SAC is below 1%, and IOT when the prevalence of any intensity of infection in SAC is 0%. We define a stochastic run to have met the target if the prevalence indicator is still below the control threshold 20 years after the end of the MDA programme.

## Results

### Reproducing initial settings

The defined initial settings of low, moderate, and high endemicity were reproducible only with a subset of models (147 out of 162). Clearly, the presence of at least one regulating mechanism is required to reproduce a stable endemic equilibrium for all three endemicity settings. In addition, adopting the model-based age-exposure function, worm- or human-level regulation alone was not sufficient to reproduce a stable, low endemic equilibrium (≤ 10% prevalence of any infection in SAC). Explicit snail population dynamics at any intensity of regulation is essential for reaching low stable endemic equilibria. With the alternative definition of age-exposure function based on water contacts we found that any regulating mechanism can initiate the model in a low endemicity setting. In particular, snail-level regulation allows very low endemic equilibria.

When the system does not reach the required endemic equilibrium, the number of simulated cercariae in the environment and the average worm load in humans can either grow without upper limits, leading quickly to an endemicity setting where the entire human population is infected, or die out, leading to a disease-free situation. Models without any regulating mechanism and models without snail-level regulation combined with the model-based exposure function are not successful in reproducing stable equilibria for all endemic settings and have therefore been excluded from the assessment of further results. Only exception to this exclusion is the model with strong worm-level regulation alone and model-based exposure function. Although this model does not allow to generate steady initial settings with low endemicity, it is included for reference in further comparisons, as current models for policy use the same assumptions.

### Reproducing observed epidemiological patterns

#### Age-intensity profiles

**Fig 2:**
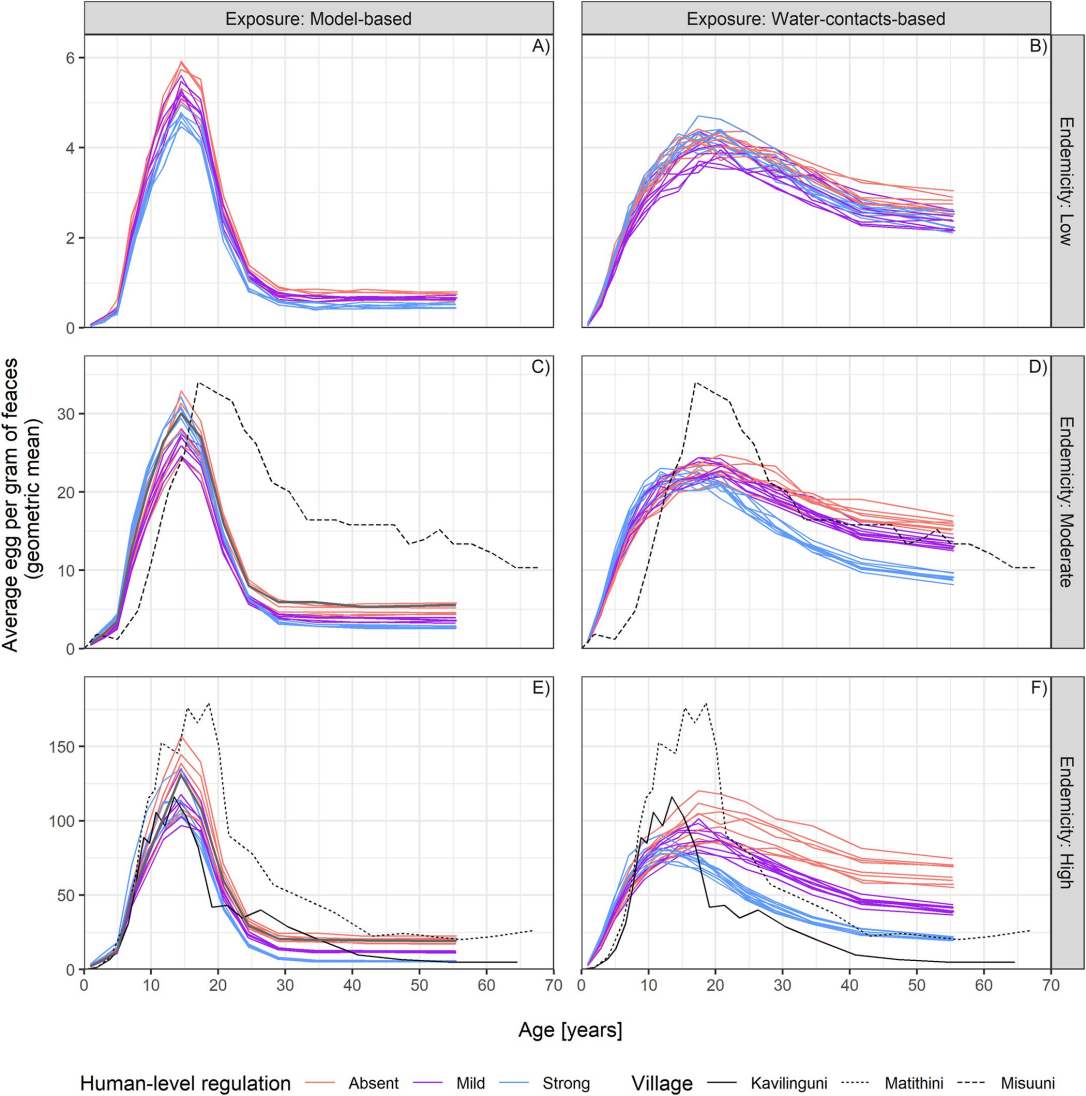
Age-intensity profiles by varying assumptions on age-exposure and anti-reinfection immunity mechanism. For each choice of the age-exposure function (columns) and different endemicity settings (rows), a single panel shows the simulated egg counts on the y-axis (mean epg is displayed, over age group and 100 stochastic realizations of the models) by age on the x-axis. The age-intensity profiles are displayed at pre-control, for all models that could successfully reproduce all three endemicity settings. Each line depicts results from a single model. The degree of regulation assumed at human level via anti-reinfection immunity is highlighted with different colours, from Absent to Strong, according to the legend in the figure. Lines of the same colour within a single panel are due to combinations (3x3 = 9) of worm- and snail-level regulation, given the same choice of immunity (colour), age-exposure function (column), and endemicity (row). Age bins are defined as to have the same number of individuals in each bin. The grey line in the left-hand-panels (C and E) represents a model with strong worm-level regulation only and the model-based exposure function. This model does not allow to generate stable initial settings with low endemicity, but it is included here for reference, as current models for policy use the same assumptions. The black lines indicate age-intensity profiles observed in three Kenyan villages and published elsewhere [53].

The assumptions on human-level regulation via anti-reinfection immunity and on the age-exposure functions have the largest impact on the resulting shape of the age-intensity profiles, whereas varying assumptions on regulation occurring at worm- and snail-level has a minor effect. **Fig 2** compares the age-intensity profiles for different degrees of regulation in humans (anti-reinfection immunity, by colours) and both age-exposure functions, for all three endemicity settings. The figure shows that the peak in intensity of infection reproduced by all models is followed by i) a steep drop for adults with the model-based exposure function, and ii) a smoother decline in adults leading to higher egg counts, using the exposure function based on water contacts. In the latter, implementing human-level regulation from absent to strong, shifts the peak from 20 to 10 years old on average and considerably decreases the mean intensity in adults. Interestingly, despite a relatively high exposure in adults assumed by the water contacts-based exposure function, a strong regulation in humans can explain the low intensity of infection in adults (20+) that is sometimes observed in specific exposure settings (**Fig 2D** and **2F**). The effect of anti-reinfection immunity on the intensity of infection is not seen in low endemicities, because the accumulation of dead parasites in human hosts is too low to trigger an immune response.

Observed age-intensity profiles from three Kenyan villages, published elsewhere [53], are plotted in **Fig 2** (black lines) for comparison with simulated profiles. The model-based exposure function can replicate age-intensity profiles in high endemicity settings reasonably well. However, at lower endemicities, the decline in intensity of infection among older ages may result in very low average epg, compared to the observed data. The alternative age-exposure function based on water contacts better reproduces different age-intensity profiles. With some degree of regulation in humans, it also replicates low intensity of infection in adults in high endemicity settings. The age-intensity profiles disaggregated for all models are displayed in **S1 Appendix.**

#### Effectiveness of MDA and prevalence bounce-back

**Fig 3:**
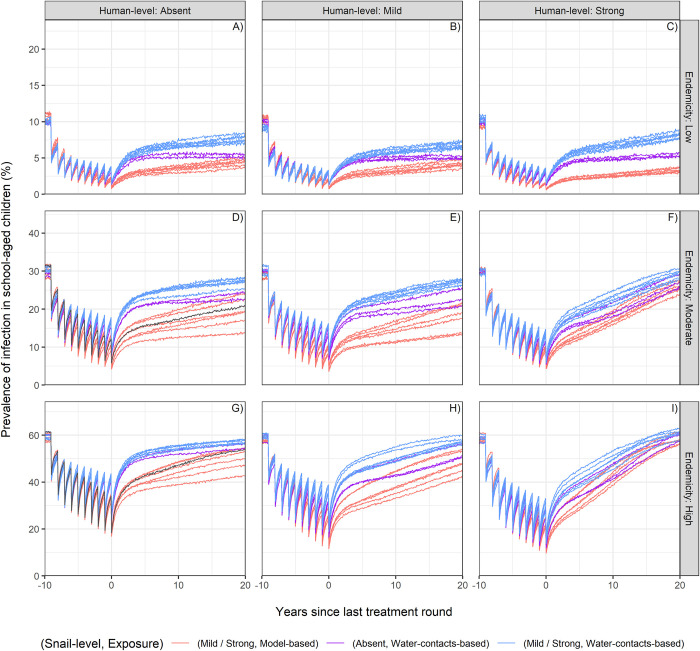
Prevalence timelines in SAC during and after treatment. For different degree of human-level regulation via anti-reinfection immunity (“Human-level”, by columns) and different endemicity settings (“Endemicity”, by rows), single panels show the infection prevalence in school-aged children on the y-axis (mean of 100 stochastic realizations of the model) by years since the last treatment round on the x-axis, for all models that could successfully reproduce all three endemicity settings. Each line depicts results from a single model. Lines of the same colour represent groups of models as indicated in the legend of the figure. “Snail-level” refers to the different assumptions of snail-level regulation. The grey line in panels D) and G) represents a model with a strong worm-level regulation only and the model-based exposure function. The model is excluded from the analyses because it does not allow stable initial settings with low endemicity, but it is added here for reference as current models for policy use the same assumptions. For all models, treatment is annually administered to 5–15 years old individuals with a coverage of 75% of the target population, 5% of target population systematically untreated, and a drug efficacy of 86%.

All models reproduce similar decreasing trends of prevalence during MDA (**Fig 3**). Assumptions on human-level regulation and the chosen age-exposure function are the main determinants of the drop in SAC prevalence due to treatment, depending on the endemicity setting. With a model-based exposure function, MDA has a stronger effect on prevalence in SAC. The prevalence drops to lower levels after 5 MDA rounds if compared to the results obtained by using the exposure function based on water contacts. This can be explained by the fact that the simulated control strategy targets the SAC group, where most of the exposure occurs, particularly when using the model-based exposure function. A strong regulation in humans also predicts a more effective treatment with respect to assuming absent or mild regulation. In fact, MDA itself contributes to the development of anti-reinfection immunity by its acting mechanisms that kills adult worms [29].

The ability of each model to reproduce the natural prevalence bounce-back is further shown in **Fig 3**, by the prevalence trends up until 20 years after the last MDA round. The figure shows that the combination of the age-exposure function based on water contacts and a mild or strong degree of regulation in snails is essential to reproduce a bounce-back of prevalence close to pre-control levels in all endemicity settings, within 20 years after termination of the MDA programme. The rationale for this result lies in a reduced impact of treatment as a consequence of the water contact-based exposure function, as previously explained, in addition to higher values of transmission parameters required if considering snail- or human-level regulation, in order to reproduce the initial endemicity settings. By assuming a strong regulation at human level in fact, the bounce-back is much more prominent, although the prevalence drops to lower values during treatment with respect to assuming the regulation to be absent or mild. The models found successful in explaining a plausible rebound of prevalence after treatment are not significantly influenced by the degree of worm-level regulation.

In conclusion, the models that were successful in explaining observed patterns (blue lines in second and third columns in **Fig 3**) were those that combined an age-exposure function based on water contacts with a mild or strong degree of regulation at both snail- and human- level. Selected successful models could explain observed patterns both in terms of pre-control age-intensity profiles and bounce-back of prevalence after treatment. The timelines of SAC prevalence during and after the MDA programme disaggregated for all models are displayed in **S2 Appendix**.

### Feasibility to reach the control targets

Despite reproducing similar epidemiological patterns, successful models can still differ in their predictions of the probability to reach the control targets. Results that follow include only such selected models, with the exception of the model with only strong worm-level regulation and model-based exposure function, as current models for policy use the same assumptions.

#### Reaching control targets with 10-year annual MDA to school-aged children

We found that all successful models agree that treating SAC only with 10 years of annual MDA will not allow reaching the EPHP in high or moderate endemicity settings. In low endemicities, we start from a setting where the prevalence of high intensity infection in SAC is already below 10% (EPHP has already been met). Here, the target of interest is the IOT and none of the models predict a positive probability to reach this target despite 10 rounds of annual MDA to SAC. Results are different for the model assuming only strong worm-level regulation and a model-based exposure function, used here as reference for common assumptions, which predicts a 73% probability to reach EPHP in a moderate endemicity setting.

#### The impact of community-wide annual MDA

**Fig 4:**
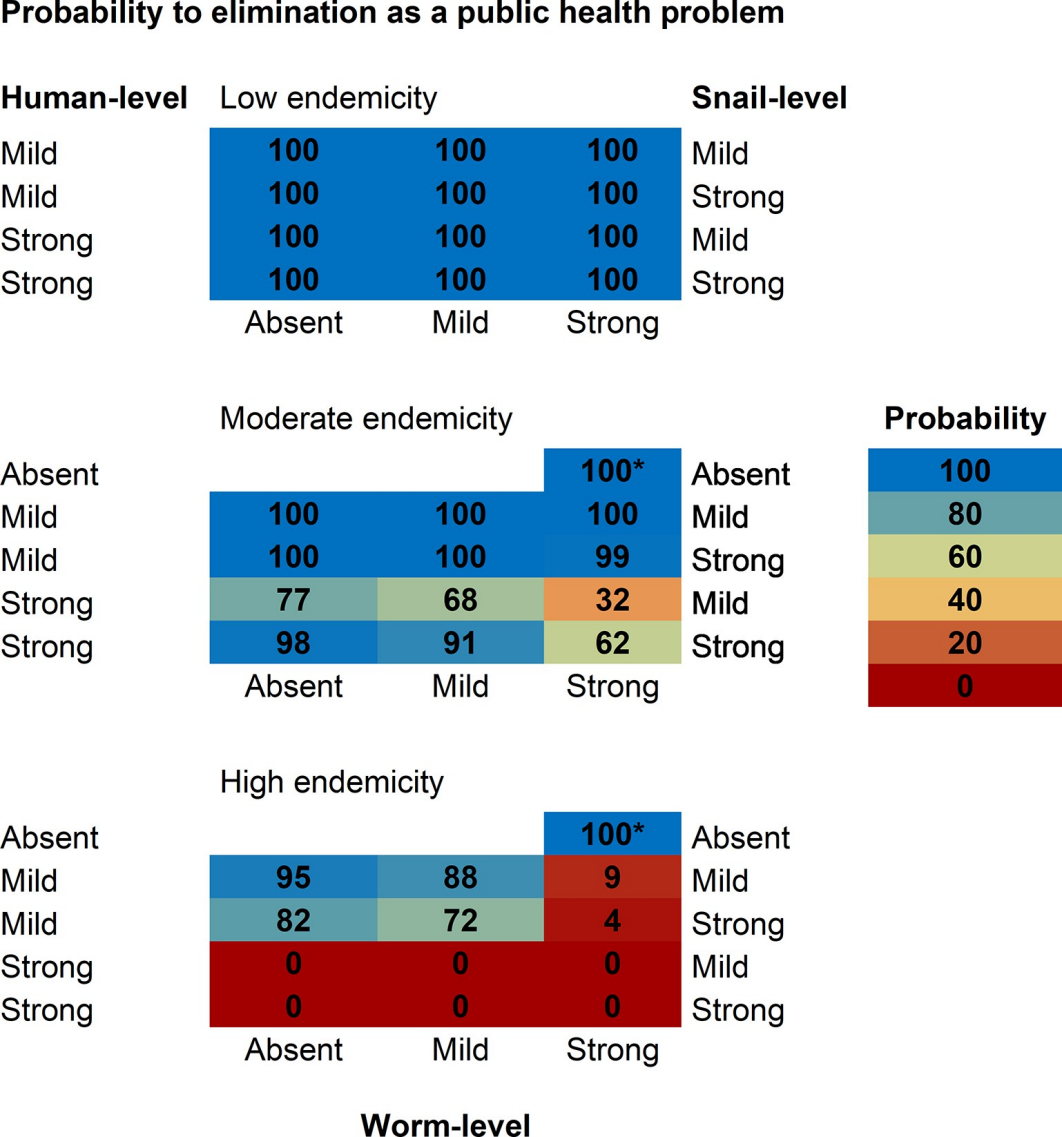
Probability to reach elimination as a public health problem with a 10-year annual MDA to all individuals older than 2. The figure reports predictions for the probability (portion of the 100 stochastic runs monitored at 20 years after end of treatment) of reaching elimination as a public health problem (EPHP, prevalence of heavy infections in school-aged children < 10%), across models successful in reproducing all observed patterns for schistosomiasis. Successful models assume an age-exposure function based on water contacts. Regulating mechanisms can be at human-level (“Human-level”, left side, via anti-reinfection immunity), snail-level (“Snail-level”, right side, via density-dependence in population growth), and worm-level (“Worm-level”, bottom, via density-dependence in egg production). *This model assumes a strong worm-level regulation only and the model-based age-exposure function. It was not successful in reproducing observed patterns within our modelling framework but it is added here for reference as current models for policy use the same assumptions. For all models, treatment is annually administered to 2+ years old individuals with a 75% coverage of the target population, 5% of target population systematically untreated, and a drug efficacy of 86%.

Expanding the 10-year annual MDA programme to all individuals older than 2 years of age, leads to EPHP in moderate endemicity settings according to all successful models with a mild human-level regulation (**Fig 4**). The mechanism of (dying-worm driven) anti-reinfection immunity (human-level regulation) has the highest impact on the probability to reach EPHP: a strong human-level regulation decreases the probability to a range between 32% and 98% if combined with another regulating mechanism (snail or worm) (**Fig 4**). In fact, in order to reproduce moderate and high endemicity settings in the presence of a strong human-level regulation, transmission rates in human hosts need to reach high values (input files with transmission parameters can be found in the public repository https://github.com/VeronicaMalizia/SchiSTOP_model). These high transmission rates cause infection prevalence to rebound more quickly compared to scenarios with mild or absent human-level regulation. Remarkably, with community-wide MDA, the treatment-induced death of worms during MDA, and the consequent stronger anti-reinfection immunity response, cannot compensate for this effect. In high endemicity settings, it is still feasible to reach EPHP with a probability ranging between 72%– 95% if human-level regulation is mild and worm-level regulation is absent or mild (**Fig 4**). The probability shows a decreasing trend with increasing degree of worm-level regulation, dropping to a range of 4% - 9% if strong worm-level regulation is assumed. In fact, strong worm-level regulating mechanism decreases here the impact of MDA in high endemicity settings, as individuals with high worm loads will still have a similar egg output after treatment because of release of density-dependent intraspecific competition, therefore keeping their contribution to transmission. Consequently, prevalence rebounds much earlier than in models without worm-level regulation (absence of density-dependency in egg production, thus a linear relationship between worm burdens and expected egg counts). According to all other models, the probability to reach EPHP is zero in high endemicity settings (**Fig 4**). The probability to reach EPHP was found to be higher (100%) when using assumptions as employed in current models used for policy (marked by *).

**Fig 5:**
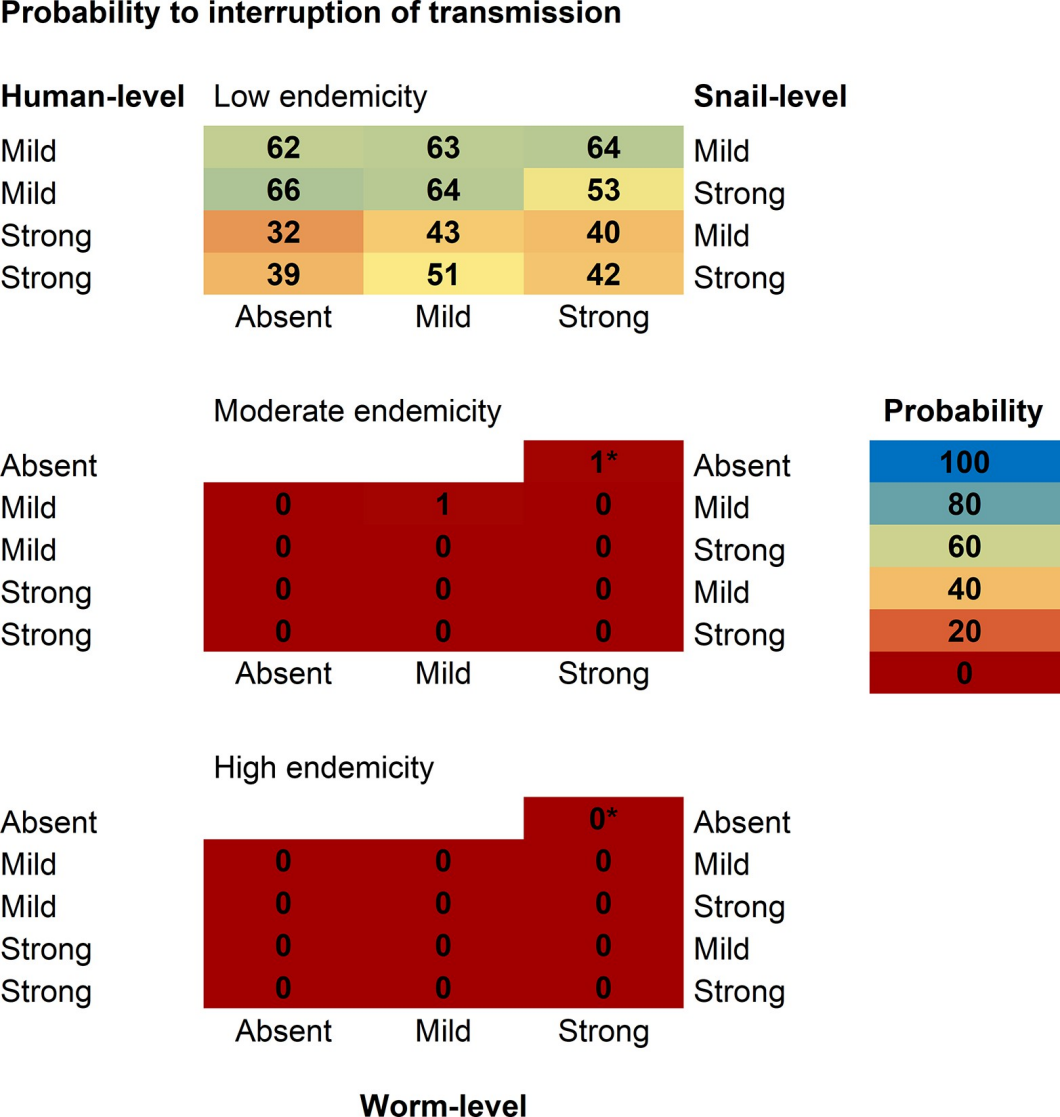
Probability to reach interruption of transmission by 10-year annual MDA to all individuals older than 2. The figure reports predictions for the probability (portion of the 100 stochastic runs monitored at 20 years after end of treatment) of reaching interruption of transmission (0% prevalence of any infection in school-aged children), across models successful in reproducing all observed patterns for schistosomiasis. Successful models assume an age-exposure function based on water contacts. Regulating mechanisms can be at human-level (“Human-level”, left side, via anti-reinfection immunity), snail-level (“Snail-level”, right side, via density-dependence in population growth), and worm-level (“Worm-level”, bottom, via density-dependence in egg production). *This model assumes a strong worm-level regulation only and the model-based age-exposure function. It was not successful in reproducing observed patterns within our modelling framework, but it is added here for reference as current models for policy use the same assumptions. For all models, treatment is annually administered to 2+ years old individuals with a 75% coverage of the target population, 5% of target population systematically untreated, and a drug efficacy of 86%.

Community-wide MDA also increases the chances of reaching IOT in low endemicity settings, with respect of only treating SAC. Here, the probability of interrupting transmission jumps from 0% (if treating only SAC) to a range of 32%– 66% across the different models (**Fig 5**).

## Discussion

The objective of our study was to revisit the modelling assumptions on the regulating mechanisms for the transmission of *S*. *mansoni* and to investigate the impact of such assumptions on the feasibility to reach the WHO control targets. To do so, we developed SchiSTOP, a new modelling framework for the transmission dynamics of *S*. *mansoni*. SchiSTOP was used to run a large number of simulations, varying the assumptions on the regulating mechanisms and the age-exposure to infection.

Overall, our results show that a degree of regulation at the snail-level and at the human-level, combined with the water contacts-based exposure function is needed to reproduce the defined endemicity settings (low, moderate, high) and to explain pre- and post-control observed patterns, i.e. the expected age-intensity profiles and a plausible rebound of prevalence after termination of an MDA programme. In particular, we found that without regulating mechanisms, the system leads quickly to settings where the entire population is highly infected, as previously demonstrated in other modelling studies [54]. Moreover, stable low endemic equilibria (prevalence of infection in SAC ≤ 10%) cannot be reproduced with the modelling assumptions on age-exposure and only worm-level regulation that are considered in current models used for policy. To our knowledge, there are no schistosomiasis modelling studies in the literature that aim to focus on low transmission settings. We found that snail-level regulation is crucial to reproduce very low stable endemic equilibria, within SchiSTOP. In fact, explicit snail dynamics with a varying carrying capacity can stabilise the system providing a continuous reservoir of infection even at very low transmission levels in humans, thanks to the force of infection acting on snails which contributes to maintain transmission.

The assumptions made on the regulating mechanisms are particularly relevant when the models are used for policy recommendations. In fact, the predicted probabilities to reach the control targets differ largely across successful models, and they are overall lower compared to the predictions obtained under the regulating assumptions of current models used for policy recommendations. Results show that according to all successful models, it is unlikely to meet EPHP and IOT when treating SAC only. If MDA is expanded to community-wide treatment of all individuals older than 2, the feasibility increases, even for IOT in low endemicity. This is in line with previous studies [55].

The present work also highlights the importance of informing the age-exposure function with water contact data. In fact, the model-based age-exposure function only performs well in reproducing age-intensity profiles in high endemicity settings, while the drop in intensity of infection at older ages may be too strong in lower endemicities. This is because this function was derived from fitting a transmission model solely considering regulation at worm-level to the age-intensity profiles from different African villages, all characterized by high endemicity [11,15,17,56,57]. Available data show that age-intensity profiles, although generally peaking in 10–20 years old, can appear differently in older ages describing heterogeneities in endemicity, exposure, and regional-specific settings [48]. The alternative age-exposure function based on water contacts effectively reproduces the variation in age-intensity profiles. Using this function, our results additionally indicate that the impact of (dying-worm driven) anti-reinfection immunity on population age-intensity profiles aligns with findings from previous studies [58] showing a peak shift to lower ages and reduced intensity in adults. We further explored the interaction of anti-reinfection immunity for *S*. *mansoni* with other regulating mechanisms and assessed their implications for control programs and expected impact of interventions. Our findings suggest that some degree of human-level regulation via (dying-worm driven) anti-reinfection immunity is necessary to explain observed age-intensity relationships in high endemicity settings. A recently published review provided evidence for water contacts being robustly associated with infection in both children and adults, across heterogeneous settings [59]. These findings shed light on the definition of at-risk populations and therefore requiring treatment and highlighted the need of standardised tools for exposure measurements. However, how to properly define and quantify exposure to infection on the basis of observable data such as number of contacts with contaminated water, duration of contact and area of the body exposed, remains a challenge.

Overall, the modelling assumptions commonly employed in schistosomiasis modelling and used for informing control strategies (i.e., in terms of age-exposure and only worm-level regulation) are found not adequate to reproduce low endemic pre-control settings and rebound of prevalence after MDA, within SchiSTOP. Predictions to reach EPHP under those assumptions are generally more optimistic compared to the same predictions for the successful models in our study. This is because i) with a model-based function, the MDA has a stronger effect on prevalence in SAC, ii) the assumption of a strong worm-level regulation requires lower transmission parameters and a higher heterogeneity of worms to represent moderate and high endemicity scenarios, compared to those required by other regulating mechanisms.

Our modelling framework, SchiSTOP, has some limitations, mainly related to the limited knowledge and reliability of data available to more precisely parametrise regulating mechanisms. In particular, we assumed that acquired immunity leads to a protective effect towards repeated infections, as suggested for *S*. *mansoni* [33]. However, the long-term dynamics of immunity still constitute a research and data gap [33]. While the current understanding points towards a slow development of immunity triggered by the death of adult worms (both naturally and by praziquantel therapy) [60], not much is known about the duration of such protection. We chose not to account for a decay of anti-reinfection immunity for the scope of these analyses. In addition, some studies suggest that the main effect of acquired immunity is a decrease in worm fecundity. This has been proposed for *S*. *haematobium* [61,62], but does not seem to be the case for *S*. *mansoni* [33,63]. The assessment of infection in snails is also subject to uncertainty due to sampling procedures, and diagnostic methods to assess infection. Field and laboratory data have shown that the detected prevalence of infected snails (thus shedding cercariae) is usually quite low [37,64–67], but exhaustive explanation of such low prevalence is lacking and no evidence of correlation between infection rates in snails and humans has been identified. We chose a fixed 6% prevalence of infection in snails to define all three endemicity initial settings. Molecular tools and genetic data are further necessary to deepen our understanding of variations in susceptibility to schistosome species by specific intermediate host snails.

Furthermore, we compute probability of IOT as the number of stochastic runs that meet the target up to 20 years after termination of the MDA programme to ensure capturing pathways of late recrudescence, despite monitoring and evaluation in the field are likely to occur within shorter time intervals [10]. A rebound after elimination, as well as maintained low transmission, can nevertheless occur via other mechanisms like movement of infected individuals [68] or cercariae/snail movement through water stream. Although human movement, particularly rural-to-urban migration, is likely to contribute to the re-emergence of the disease through importation of infection [69], SchiSTOP does not take movement of individuals into account. We assume a close population reproducing the size of a rural village, and we consider human movement to play a minor role for schistosomiasis, given its very focal nature [3].

We assume treatment by MDA to kill 86% of adult worms, in line with other existing models [17,21,40,70]. This value originates from a field study [71] where drug efficacy is determined by the observed egg reduction rate (ERR) due to praziquantel, defined as the relative reduction in the group mean egg output after treatment compared to pre-treatment levels [72]. The observed ERR does not translate directly into the worm killing rate because of the high individual variation in egg counts, and this difference is dependent on our assumptions on worm-level regulation. However, our analysis shows that the 86% reduction in adult worms results in a mean ERR (based on single Kato-Katz slide) after one round of MDA that varies in a small range (between 81% and 83% across absent, mild, and strong worm-level regulation). It is important to mention that the sampling methods used for the field assessment of ERR likely lead to an overestimation of efficacy due to the fact that egg-negative individuals at baseline are excluded, but light infections might have missed [73]. Therefore, we chose a constant portion of 86% of worms killed by MDA, for the scope of our analysis and we performed a sensitivity analysis by varying such parameter between 80% and 90% (**S3 Appendix**). Even in the case of 90% of worms killed by MDA, none of the successful models predict a probability to reach the control targets greater than 0 when treating only SAC. In the case of community-wide treatment instead, although absolute predictions to achieve control targets vary according to a higher or lower worm killing rate, the trend across regulating scenarios remains unchanged.

SchiSTOP integrates an ODE module to describe the explicit snail dynamics into an ABM framework capturing dynamics and complexities of transmission in the human host. SchiSTOP considers limited snail population growth, in line with commonly-made assumptions of resource competition [36]. The depletion of susceptible snails and increased mortality of infected snails are thus responsible of regulating transmission. Other aspects in snail transmission dynamics have been considered in the literature and have been shown to play an important role in transmission on humans, and therefore in the expected impact of interventions, such as MDA. Some examples are: the effect of seasonality, temperature and annual rainfalls [34], snail age structure [74], and saturating force of infection for snails [35]. Here, we mainly focus on exploring the role of snail dynamics in interaction with other regulating mechanisms such as (dying-worm driven) anti-reinfection immunity and density-dependence in egg production.

In this work, SchiSTOP has been used to revisit the assumptions of regulating mechanisms for *S*. *mansoni* transmission. However, its formulation is suitable to answer diverse research questions about the epidemiology and control of schistosomiasis. For instance, the presence of a specific module for the dynamics in snails allows for a thorough assessment of the impact of snail control interventions.

In conclusion, the present work highlights the importance of considering regulating mechanisms at different levels of the transmission cycle in models for schistosomiasis transmission and control, as they largely determine the predicted impact of interventions. We showed that some degree of regulation in both snail- and human-level is required to explain commonly observed epidemiological patterns for schistosomiasis. Our findings support the current WHO guidelines, recommending community-wide treatment to all individuals > 2 years old. This control strategy greatly enhances the probability to reach the control targets, also in high endemicity settings. However, the probability to reach these targets is likely not as high as predicted by the currently used models that only assume worm-level regulation. We stress on the need for a better understanding of the mechanisms regulating transmission and persistence of schistosomiasis in endemic settings.

## Supporting information

S1 Text Full model specification: SchiSTOP. (PDF)

S1 Table Complete list of parameters employed for the analyses. (PDF)

S1 Appendix Age-intensity profiles for the complete set of models. For each choice of the age-exposure function and each endemicity setting (titles), single panels refer to a given combination of the assumptions of worm-level regulation via density-dependence in egg production (“Worm-level”, columns) and snail-level regulation via explicit snail modelling (“Snail-level”, rows). Each line depicts results from a single model. The degree of regulation assumed at human level via anti-reinfection immunity (“Human-level”) is highlighted with different colours, from Absent to Strong, according to the legend above each figure. A single panel shows the simulated egg counts on the y-axis (mean epg is displayed, over age group and 100 stochastic realizations of the model) by age on the x-axis. The age-intensity profiles are displayed at stable pre-control settings, by varying the degree of regulating mechanism in humans (colours). Age bins are defined as to all be equally sized.
(PDF)

S2 Appendix Effectiveness of treatment and prevalence bounce-back for the complete set of models. For each choice of the age-exposure function and endemicity setting (titles), single panels refer to a fixed combination of the assumptions of human-level regulation via anti-reinfection immunity ("Human-level", columns) and snail-level regulation via explicit snail modelling ("Snail-level", rows). The degree of regulation assumed at worm level density-dependence in egg production ("Worm-level") is highlighted with different colours, from Absent to Strong, according to the legend above each figure. A single panel shows the infection prevalence in school-aged children on the y-axis (mean of 100 stochastic realizations of the model, single runs as shaded lines) by round of treatment on the x-axis. For all models, treatment is annually administered to 5–15 years old individuals with 10 repeated rounds, a coverage of 75% of the target population, 5% of target population systematically untreated, and a drug efficacy of 86%.
(PDF)

S3 Appendix Sensitivity analysis varying drug efficacy. (PDF)
