# Supplementary material for: Revisiting the impact of Schistosoma mansoni regulating mechanisms on transmission dynamics using SchiSTOP, a novel modelling framework
Source: PLoS Negl Trop Dis. 2024 Sep 20;18(9):e0012464. doi: 10.1371/journal.pntd.0012464 (PMC11414988; doi:10.1371/journal.pntd.0012464)
Supplement: S1 Text — (PDF) [file pntd.0012464.s001.pdf]

Supporting information for "Revisiting the impact of *Schistosoma mansoni* regulating mechanisms on transmission dynamics using SchiSTOP, a novel modelling framework"

Veronica Malizia[1,2], Sake J. de Vlas[1], Kit C.B. Roes[2], Federica Giardina[2]

1 Department of Public Health, Erasmus MC, University Medical Center Rotterdam, Rotterdam, The Netherlands

2 IQ Health Department, Biostatistics Research Group, Radboud Institute for Health Sciences, Radboud University Medical Center, Nijmegen, The Netherlands

Introduction

This document contains full specification of the modelling framework SchiSTOP, including functions and parameters employed for the simulations. We implemented an agent-based stochastic model (ABM) to reproduce the transmission dynamics of schistosomiasis between the human hosts and the contaminated water environment, via larvae multiplication in the intermediate host, namely freshwater snails. The dynamics of the snail population are explicitly included via a deterministic model consisting of a system of ordinary differential equations (ODEs), integrated into the ABM. The five main building blocks of SchiSTOP are: the human population, the parasitic worms living in the human host, the two parasitic larval stages living in the contaminated water environment, and the snail population.

The ABM is based on stochastic events updated at discrete time steps t of 1 month. The ODEs system is defined at continuous time scale and a daily time step t'. Events are governed by daily rates, considering the short lived larval stages of *Schistosoma mansoni*. At each time step t of the ABM, we simulate from the system of ODEs over a time horizon h equal to 1 month. Afterwards, the results from these simulations serve as initial conditions for the next ABM-time step $t + 1$.

The model is written in R programming language, version 4.2.2 [1] and the code is available for consultation and downloading at the public online repository `https://github.com/VeronicaMalizia/SchiSTOP_model`.

Here on, we refer the subscript i to the i-th human individual alive at time t. The human population at each time step t is tracked as a matrix $N \times m$ where N is the number of alive individuals (i.e., the current population size) and m the columns storing the following individual characteristics: age, sex, individual susceptibility to infection, parasite acquisition rate, number of juvenile worms, number of adult worm pairs, cumulative dead worm pairs, and observed egg counts. All parameters employed in the model and mentioned in the next sections of this document are listed and documented in Table 1.

Human demography

The human population is governed by births, deaths, aging, and migration. The human demography is parameterised to follow the age-distribution in Uganda as reported by the Ugandan bureau of statistics [2].

The number of newborn individuals is deterministically obtained with a fixed birth rate. People at birth have zero worm/egg loads and their sex is assigned as a random draw with probability 0.5.

Individuals migrate according to a fixed monthly net migration rate, which is tuned to reach an equilibrium population size of $N = 1000$, assuming the model replicates the transmission dynamics in a rural community. Individuals of age between 5 and 55 years are eligible for migration.

Each month individuals can die according to an age- and sex- specific death probability. In case of death, the individual is removed from the population matrix.

The population is accordingly updated and aged of a period equal to t. The demographic dynamics are run to the equilibrium for 200 years and the final age distribution is used to initialise the transmission model (Fig 1).

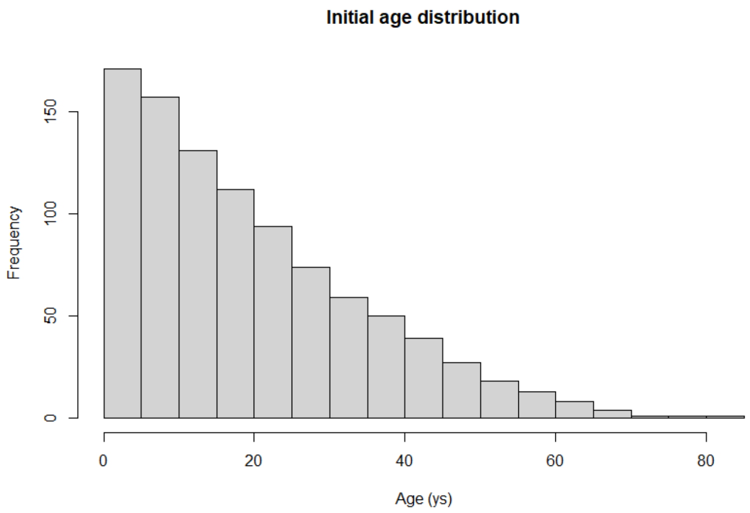

Fig 1. Initial age distribution. The histogram represents the age distribution used to run the simulations for the present work. This distribution is extracted after running the demographic dynamics to the equilibrium with no presence of infection dynamics, for a total of 200 years. The human demography is parameterised to follow the age-distribution of Uganda as estimated by the Ugandan bureau of statistics [2].

Infection dynamics

The transmission cycle of *S. mansoni* occurs between the human host and the contaminated water environment, mediated by freshwater snails acting as intermediate host. The first-stage larvae (free-living miracidia) are released from eggs hatching upon

contact with freshwater, which search for certain species of freshwater snails. Asexual reproduction occurs in the snails, leading to the release of second-stage larvae (free-living cercariae). These cercariae can infect humans, where they mature into adult worms, pair and reproduce sexually. Individuals infected with *S. mansoni* contribute to the water environment with their excreta containing parasite eggs, thereby maintaining transmission.

Human exposure to infection

The force of infection acting on each human host is defined as follows:

$$FOI_{H_i}(t) = \phi_i(t)\ \exp(-\alpha_{imm} d_i(t))$$

where $\phi_i(t)$ describes the parasite acquisition rate, multiplied by an exponential factor describing the effect of human-level regulation implemented as (dying-worm driven) anti-reinfection immunity: this dictates the level of protection from consecutive reinfections. The immunity coefficient α_{imm} is a function of the dead adult worm pairs accumulated up to the current time step ($d_i(t)$). The value assumed for the immunity coefficient α_{imm} modulates the degree of the regulating mechanism in humans.

The parasite acquisition rate ϕ in each human host is defined as a function of the current amount of cercariae in the environment ($C(t)$), the overall transmission parameter on humans ζ and the relative exposure to infection x_i:

$$\phi_i(t) = \frac{C(t)\ \zeta\ x_i}{\sum_{i=1}^{N} x_i}$$

The individual relative exposure to infection is $x_i = Ex\,(a_i)\,s_i$, where:

$Ex\,(a_i)$ is the relative exposure of an individual with age a, defined as a piece-wise function of user-defined exposure rates, for a finite set of ages. This incorporates the age-specific pattern of exposure to the contaminated water;

s_i: individual susceptibility to infection, which captures personal factors influencing the chance to get exposed to infection, e.g., contacts with water due to occupation. This parameter is assumed to follow a gamma distribution with mean 1.0 and shape and rate (or 1/scale) equal to k_w. The individual susceptibility of a person is assigned at birth and remains constant throughout lifetime.

Subsequently, each human host acquires new juvenile worms ($j_i(t)$) according to a Poisson process with a rate given by the $FOI_{H_i}(t)$. The process of worms acquisition is therefore a Poisson-Gamma mixture, namely the individual worm burden follows a negative binomial distribution with aggregation parameter k_w. Such distribution has been shown to be adequate for describing the overdispersion of worms around the mean [3].

Newly acquired worms are assumed to be juveniles for a given pre-patent period. Juvenile worms do not mate nor reproduce. Mortality of juvenile worms is neglected. Once the pre-patent period is over, juvenile worms are randomly assigned sex according to a fixed probability of 0.5 and considered mature and patent to pair and reproduce.

Worm life within the human host

In the model, mature worms live in pairs. Individual worm pairs ($w_i(t)$) are defined as the minimum number between female and male juvenile worms (all possible pairs are formed). Worms which do not pair will not survive the next time step. In the rest of the transmission cycle, mature worm pairs are considered as infective units for simplicity, supported by the monogamous nature of *Schistosoma*. Similarly to the assumptions used by previous models [4], the adult worms' lifespans follow an Erlang distribution with a rate r and a shape equal to a specified number of stages throughout which the worm pairs are distributed within each human host. The newly paired worms enter the first stage and are subsequently aged through all the remaining stages before they die. In each stage, the portion of worm pairs aging to the next step is given by $\psi = e^{-r} = e^{-(s/T_w)}$, hence assuming exponential survival within each stage. Here, s is the number of considered stages and T_w the adult worm lifespan.

Worm pairs are expected to reproduce and to pass eggs into the human intestine. The eggs will be released into the environment through the faeces. The model accounts for egg detection via the Kato-Katz diagnostic test, that uses stool smear samples of 41.7 mg for egg counting. Assuming that all paired worms reproduce within a time step of one month, we define the individual expected number of eggs observed in a stool sample as: $\mu_i\,(t) = \ \alpha\ w_i(t)\ e^{-z\ w_i(t)}$. Here, the parameter α represents the fecundity parameter (i.e. the expected number of eggs / worm pair / stool sample, in absence of density dependence in egg production) and z is a density-dependency factor regulating the level of saturation in egg production, therefore tuning the regulating mechanism at worm level. In a modelling setting without regulation occurring at worm level (meaning in absence of density-dependence in egg production), we set $z = 0$ assuming the produced egg load to linearly depend on the number of worm pairs.

Diagnostic test

SchiSTOP simulates results of a single Kato-Katz diagnostic tests, at pre-defined moments along the simulation. The result of the diagnostic test is drawn from a negative binomial distribution with mean equal to $\mu_i(t)$ at the current time step, and aggregation parameter k_e. If multiple tests by individual are desired by the user, the results are averaged per person.

Human contribution to the water environment

The number of eggs (e_i) by which each individual contributes to the water environment is given by multiplying the quantity ($\mu_i \times 24$), which translate into eggs per gram of

feaces, by the daily average of excreted quantity of faeces expressed in grams. We define the individual contributions (o_i) as:

$$o_i\left(t\right) = e_i\left(t\right)\ Con(a_i)$$

where $Con(a_i)$ indicates the relative contribution of an individual with age a, defined as a piece-wise function of user-defined contribution rates, for a finite set of ages. This accounts for any age-specific pattern in human contribution to infection. For the scope of the current work, we set a flat relative contribution function, that we assume to be independent on age.

We assume that all excreted eggs hatch in the water and mature into miracidia in a negligible simulation time. We can therefore immediately update the total miracidial uptake into the water environment $M(t)$ as the sum of individual contributions:

$$M\left(t\right) = \sum_{i=1}^{N} o_i(t)$$

Intermediate host

Implicit intermediate snail host

In the simplest model formulation, no regulating mechanism is assumed to act at snail level. In such model variant, the dynamics in the intermediate host are implicitly modelled as maturation of miracidia into cercariae. The miracidia released in the water environment will simply mature into cercariae in a period h which approximates the maturation period within the intermediate host, i.e., $C\left(t\right) = M\left(t - h\right)$.

Explicit intermediate snail host

SchiSTOP allows explicit modelling of the intermediate host, by means of a separate module for the simulation of infection dynamics within the snail population. The module consists of a deterministic equation-based model that represents the role of snails and their interaction with the human population.

The deterministic module is implemented as a compartmental model [5] where births, deaths, exposure, and infection of snails occur. Finally, cercariae are shed in the water environment. The course of events is governed by daily rates. See Fig2 for a schematic representation of the snail transmission dynamics. The state of the system $(S\left(t'\right),\ E\left(t'\right),\ I\left(t'\right),\ C(t'))$ describes in order: the amount of susceptible, exposed, infected snails, and cercariae available at ODE-time step t'. The dynamics between compartments are described by the following system of ordinary differential equations with given initial conditions.

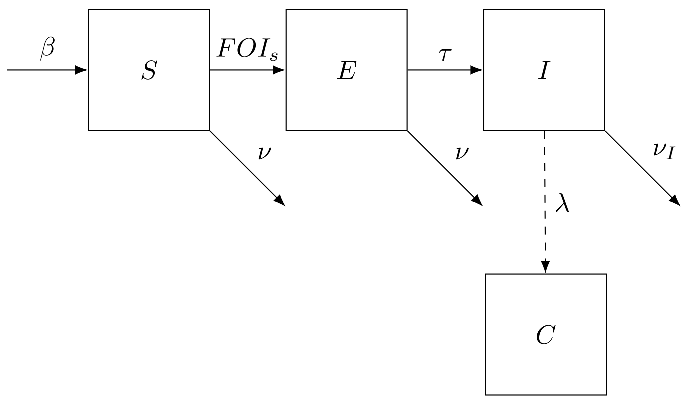

Fig 2. Compartmental module simulating the transmission dynamics in the snail population and the cercarial production.

$$
\begin{aligned}
\frac{dS(t')}{dt'} &= \beta - (\nu + FOI_s(t))\ S(t') \\
\frac{dE(t')}{dt'} &= FOI_s(t)\ S(t') - (\nu + \tau)\ E(t') \\
\frac{dI(t')}{dt'} &= \tau\ E(t') - \nu_I\ I(t') \\
\frac{dC(t')}{dt'} &= \lambda\ I(t') - \gamma\ C(t')
\end{aligned}
$$

Newly born snails are susceptible to schistosomiasis infection. Susceptible snails reproduce with a birth rate $\beta(t')$, according to a logistic growth due to competition for resources:

$$\beta(t') = \beta_0\ \left(1 - \frac{N_s(t')}{K}\right)\ (S(t') + E(t'))$$

Here, β_0 is the maximum reproduction rate in absence of competition, $N_s\,(t')$ the total snail population size, and K the carrying capacity. K is tuned to parameterise a mild or strong degree of snail-level regulating mechanism. Only susceptible $S(t')$ and exposed $E\,(t')$ snails are assumed to contribute to reproduction, due to castration upon parasitic infection.

Susceptible snails can encounter invasion of miracidia released in the water environment after egg excretion by human hosts and become exposed according to a linear force of infection $FOI_s(t) = \eta\ M(t)$ where η is the transmission parameter on snails. Since the miracidial input $M(t)$ is a quantity obtained by the ABM simulation, the $FOI_s(t)$ is considered a constant parameter within the time horizon of the compartmental module.

Miracidia live in exposed snails for a maturation period h, after which cercariae are released. Susceptible and exposed snails undergo natural mortality, according to a mortality rate ν. When the parasites are patent, the hosting snails move to the infected

stage $I(t')$ with a rate $\tau = \frac{1}{h}$. Infected snails experience increased mortality (ν_I) and castration. Infected snails shed cercariae ($C(t')$) into the environment, according to a cercarial per capita production rate λ. Cercariae can naturally die with a rate γ before infecting humans.

At each ABM-time step t, the ODEs module is run for a period equal to h, with the aim to approximate the miracidial maturation in the intermediate host. The state $(S(h), E(h), I(h))$ is then set as initial conditions for the ODE system at the following ABM-time step $t+1$. Similarly, the cercarial amount is updated as $C(t+1) := C(h)$ and it will contribute to the definition of the $FOI_H(t+1)$.

Mass drug administration

SchiSTOP allows the incorporation of control activities such as Mass drug administration (MDA). MDA with praziquantel is implemented as a process that causes the death of adult worms (with a certain probability) in the treated individuals. Several parameters can be varied to reflect different options of treatment campaigns and efficacy.

- Length, namely how many years the MDA campaign last for
- Frequency, in terms of how often MDA campaigns implemented e.g. every second year, every year, twice a year
- Target population eligible for the treatment, e.g. School-aged children only or the whole community
- Coverage: portion of the target population actually reached by the treatment
- Target population systematically untreated: fraction of the target population that is not reached by MDA over consecutive rounds
- Drug efficacy: fraction of killed adult worms (pairs) per each human host

It is important to note that only adult worms die with praziquantel. Killed worms contribute to the amount of accumulated dead worms that in turn act as a trigger for the development of anti-reinfection immunity in our model.

Table 1: **Parameters employed in the model.**

Parameter	Value	Source
**Human demography**		
Population size (N)	1000	Assumption
Birth rate [annual, per 1000 individuals]	36.5	[6]
Emigration rate [annual, per 1000 individuals]	18.6	Tuned
Eligible age group for migration [years old]	$[5-55]$	[2]
Death probabilities by age	$[0-1]$	[6] and avail. at SchiSTOP
**Parasite life within human host**		
Aggregation of worms ($k_w > 0$)	-	Available at SchiSTOP
Transmission parameter on humans ($\zeta > 0$)	-	Available at SchiSTOP
Age specific relative exposures	*Model-based*: Piece-wise constant. (0.032, 0.61, 1, 0.06) for ages (0-4, 5-9, 10-15, 16+).<br>*Based on water contacts*: Piece-wise linear. (0, 0.62, 1, 0.51, 0.51) for ages (0, 5, 15, 40+).	[7–10]
External force of infection	Value = [0.5 - 5] worms<br>Duration = [0.5 - 2] years	Assumption
Average lifespan of adult worms within the human host [months]	60	[11]
Pre-patent period [constant, months]	3	[11]
**Egg production**		
Expected number of eggs per sample ($\alpha > 0$) [eggs/worm pair/sample]	[0.12 - 0.14]	[12,13] and **Methods** main text

Continued on next page

Table 1: **Parameters employed in the model.** (Continued)

Parameter	Value	Source
Density dependence in egg production (z) [/ worm pair]	(Absent) 0, (Mild) 0.00022, (Strong) 0.0007	[4] and **Methods** main text
Daily grams of stool produced by each human individual [gr]	150	[13]
Aggregation of observed egg counts ($k_e > 0$)	0.87	[13]
**Anti-reinfection immunity**		
Immunity coefficient ($\alpha_{imm} > 0$)	(Absent) 0, (Mild) 0.0005, (Strong) 0.002	[14] and **Methods** main text
**Snail population module**		
Maximum reproduction rate ($\beta_0 > 0$) [1 / days]	1	[5]
Carrying capacity ($k > 0$) [number of snails]	(Absent) -, (Mild) 20000, (Strong) 10000	Varying
Natural mortality of snails ($\nu > 0$)	$v = \frac{1}{100\ days}$	[5]
Mortality of snails upon infection ($\nu_I > 0$)	$v = \frac{1}{30\ days}$	[5]
Snail transmission parameter ($\eta > 0$)	-	Available at SchiSTOP
Worm maturation period within the snail ($h > 0$) [days]	30	[4, 15]
Cercarial production rate ($\lambda > 0$) [1/days]	50	[5]
Mortality rate of cercariae ($\gamma > 0$) [1/days]	1	[5]
**Mass drug administration**		
Target population	$5 - 15$ or 2+ years old	Assumption
Duration	10 years	Assumption
Frequency	Annual	Assumption

Continued on next page

Table 1: **Parameters employed in the model.** (Continued)

**Parameter**	**Value**	**Source**
Coverage (% of target population reached by treatment)	75%	[16]
Efficacy (% of killed adult worm pairs)	86%	[8]
Fraction systematically untreated	5% of target population	[8]

References

1. R Core Team. R: A language and environment for statistical computing.; 2022. Available from: https://www.R-project.org/.

2. (UBOS) UBoS. Uganda National Household Survey 2019/2020. UBOS; 2021.

3. Bradley DJ, May RM. Consequences of helminth aggregation for the dynamics of schistosomiasis. Trans R Soc Trop Med Hyg. 1978;72(3):262–273.

4. Graham M, Ayabina D, Lucas TC, Collyer BS, Medley GF, Hollingsworth TD, et al. SCHISTOX: An individual based model for the epidemiology and control of schistosomiasis. Infect Dis Model. 2021;6:438–447.

5. Civitello DJ, Angelo T, Nguyen KH, Hartman RB, Starkloff NC, Mahalila MP, et al. Transmission potential of human schistosomes can be driven by resource competition among snail intermediate hosts. PNAS. 2022;119(6):e2116512119.

6. World Health Organization. The global health observatory; 2022. Available from: https://www.who.int/data/gho/data/themes/topics/indicator-groups/indicator-group-details/GHO/gho-ghe-global-health-estimates-life-tables.

7. Toor J, Turner HC, Truscott JE, Werkman M, Phillips AE, Alsallaq R, et al. The design of schistosomiasis monitoring and evaluation programmes: The importance of collecting adult data to inform treatment strategies for Schistosoma mansoni. PLoS Negl Trop Dis. 2018;12(10):e0006717.

8. Turner HC, Truscott JE, Bettis AA, Farrell SH, Deol AK, Whitton JM, et al. Evaluating the variation in the projected benefit of community-wide mass treatment for schistosomiasis: Implications for future economic evaluations. Parasit Vectors. 2017;10(1):213.

9. Sow S, de Vlas SJ, Stelma F, Vereecken K, Gryseels B, Polman K. The contribution of water contact behavior to the high Schistosoma mansoni Infection rates observed in the Senegal River Basin. BMC Infect Dis. 2011;11(1):198.

10. Fulford AJC, Ouma JH, Kariuki HC, Thiongo FW, Klumpp R, Kloos H, et al. Water contact observations in Kenyan communities endemic for schistosomiasis: methodology and patterns of behaviour. Parasitol. 1996;113(3):223–241.

11. Anderson RM, May RM. Helminth infections of humans: mathematical models, population dynamics, and control. Adv parasitol. 1985;24:1–101.

12. de Vlas SJ, Van Oortmarssen GJ, Gryseels B, Polderman AM, Plaisier AP, Habbema JD. SCHISTOSIM: a microsimulation model for the epidemiology and control of schistosomiasis. Am J Trop Med Hyg. 1996;55(5 Suppl):170–175.

13. de Vlas SJ, Gryseels B, Van Oortmarssen G, Polderman A, Habbema J. A model for variations in single and repeated egg counts in Schistosoma mansoni infections. Parasitol. 1992;104(3):451–460.

14. Chan MS, Mutapi F, Woolhouse MEJ, Isham VS. Stochastic simulation and the detection of immunity to schistosome infections. Parasitol. 2000;120(2):161–169.

15. Gurarie D, King CH, Yoon N, Li E. Refined stratified-worm-burden models that incorporate specific biological features of human and snail hosts provide better estimates of Schistosoma diagnosis, transmission, and control. Parasit Vectors. 2016;9(1):428.

16. World Health Organization. WHO guideline on control and elimination of human schistosomiasis. Geneva: WHO; 2022.
