# Supplementary material for: Revisiting the impact of Schistosoma mansoni regulating mechanisms on transmission dynamics using SchiSTOP, a novel modelling framework
Source: PLoS Negl Trop Dis. 2024 Sep 20;18(9):e0012464. doi: 10.1371/journal.pntd.0012464 (PMC11414988; doi:10.1371/journal.pntd.0012464)
Supplement: S1 Table — (PDF) [file pntd.0012464.s002.pdf]

Table S1: **Parameters employed in the model.**

**Parameter**	**Value**	**Source**
**Human demography**		
Population size (N)	1000	Assumption
Birth rate [annual, per 1000 individuals]	36.5	[1]
Emigration rate [annual, per 1000 individuals]	18.6	Tuned
Eligible age group for migration [years old]	$[5 - 55]$	[2]
Death probabilities by age	$[0 - 1]$	[1] and avail. at SchiSTOP
**Parasite life within human host**		
Aggregation of worms ($k_w > 0$)	-	Available at SchiSTOP
Transmission parameter on humans ($\zeta > 0$)	-	Available at SchiSTOP
Age specific relative exposures	*Model-based*: Piece-wise constant. (0.032, 0.61, 1, 0.06) for ages (0-4, 5-9, 10-15, 16+). *Based on water contacts*: Piece-wise linear. (0, 0.62, 1, 0.51, 0.51) for ages (0, 5, 15, 40+).	[3–6]
External force of infection	Value = [0.5 - 5] worms<br>Duration = [0.5 - 2] years	Assumption

Continued on next page

Table S1: **Parameters employed in the model.** (Continued)

Parameter	Value	Source
Average lifespan of adult worms within the human host [months]	60	[7]
Pre-patent period [constant, months]	3	[7]
**Egg production**		
Expected number of eggs per sample ($\alpha > 0$) [eggs/worm pair/sample]	[0.12 - 0.14]	[8,9] and **Methods** main text
Density dependence in egg production (z) [/ worm pair]	(Absent) 0, (Mild) 0.00022, (Strong) 0.0007	[10] and **Methods** main text
Daily grams of stool produced by each human individual [gr]	150	[9]
Aggregation of observed egg counts ($k_e > 0$)	0.87	[9]
**Anti-reinfection immunity**		
Immunity coefficient ($\alpha_{imm} > 0$)	(Absent) 0, (Mild) 0.0005, (Strong) 0.002	[11] and **Methods** main text
**Snail population module**		
Maximum reproduction rate ($\beta_0 > 0$) [1 / days]	1	[12]
Carrying capacity ($k > 0$) [number of snails]	(Absent) -, (Mild) 20000, (Strong) 10000	Varying
Natural mortality of snails ($\nu > 0$)	$v = \frac{1}{100\ days}$	[12]
Mortality of snails upon infection ($\nu_I > 0$)	$v = \frac{1}{30\ days}$	[12]

Continued on next page

Table S1: **Parameters employed in the model.** (Continued)

**Parameter**	**Value**	**Source**
Snail transmission parameter ($\eta > 0$)	-	Available at SchiSTOP
Worm maturation period within the snail ($h > 0$) [days]	30	[10,13]
Cercarial production rate ($\lambda > 0$) [1/days]	50	[12]
Mortality rate of cercariae ($\gamma > 0$) [1/days]	1	[12]
**Mass drug administration**		
Target population	$5 - 15$ or 2+ years old	Assumption
Duration	10 years	Assumption
Frequency	Annual	Assumption
Coverage (% of target population reached by treatment)	75%	[14]
Efficacy (% of killed adult worm pairs)	86%	[4]
Fraction systematically untreated	5% of target population	[4]

References

1. World Health Organization. The global health observatory; 2022. Available from: https://www.who.int/data/gho/data/themes/topics/indicator-groups/indicator-group-details/GHO/gho-ghe-global-health-estimates-life-tables.

2. (UBOS) UBoS. Uganda National Household Survey 2019/2020. UBOS; 2021.

3. Toor J, Turner HC, Truscott JE, Werkman M, Phillips AE, Alsallaq R, et al. The design of schistosomiasis monitoring and evaluation programmes: The importance of collecting adult data to inform treatment strategies for Schistosoma mansoni. PLoS Negl Trop Dis. 2018;12(10):e0006717.

4. Turner HC, Truscott JE, Bettis AA, Farrell SH, Deol AK, Whitton JM, et al. Evaluating the variation in the projected benefit of community-wide mass treatment for schistosomiasis: Implications for future economic evaluations. Parasit Vectors. 2017;10(1):213.

5. Sow S, de Vlas SJ, Stelma F, Vereecken K, Gryseels B, Polman K. The contribution of water contact behavior to the high Schistosoma mansoni Infection rates observed in the Senegal River Basin. BMC Infect Dis. 2011;11(1):198.

6. Fulford AJC, Ouma JH, Kariuki HC, Thiongo FW, Klumpp R, Kloos H, et al. Water contact observations in Kenyan communities endemic for schistosomiasis: methodology and patterns of behaviour. Parasitol. 1996;113(3):223–241.

7. Anderson RM, May RM. Helminth infections of humans: mathematical models, population dynamics, and control. Adv parasitol. 1985;24:1–101.

8. de Vlas SJ, Van Oortmarssen GJ, Gryseels B, Polderman AM, Plaisier AP, Habbema JD. SCHISTOSIM: a microsimulation model for the epidemiology and control of schistosomiasis. Am J Trop Med Hyg. 1996;55(5 Suppl):170–175.

9. de Vlas SJ, Gryseels B, Van Oortmarssen G, Polderman A, Habbema J. A model for variations in single and repeated egg counts in Schistosoma mansoni infections. Parasitol. 1992;104(3):451–460.

10. Graham M, Ayabina D, Lucas TC, Collyer BS, Medley GF, Hollingsworth TD, et al. SCHISTOX: An individual based model for the epidemiology and control of schistosomiasis. Infect Dis Model. 2021;6:438–447.

11. Chan MS, Mutapi F, Woolhouse MEJ, Isham VS. Stochastic simulation and the detection of immunity to schistosome infections. Parasitol. 2000;120(2):161–169.

12. Civitello DJ, Angelo T, Nguyen KH, Hartman RB, Starkloff NC, Mahalila MP, et al. Transmission potential of human schistosomes can be driven by resource competition among snail intermediate hosts. PNAS. 2022;119(6):e2116512119.

13. Gurarie D, King CH, Yoon N, Li E. Refined stratified-worm-burden models that incorporate specific biological features of human and snail hosts provide better estimates of Schistosoma diagnosis, transmission, and control. Parasit Vectors. 2016;9(1):428.

14. World Health Organization. WHO guideline on control and elimination of human schistosomiasis. Geneva: WHO; 2022.
