# Supplementary material for: Revisiting the impact of Schistosoma mansoni regulating mechanisms on transmission dynamics using SchiSTOP, a novel modelling framework
Source: PLoS Negl Trop Dis. 2024 Sep 20;18(9):e0012464. doi: 10.1371/journal.pntd.0012464 (PMC11414988; doi:10.1371/journal.pntd.0012464)
Supplement: S1 Appendix — For each choice of the age-exposure function and each endemicity setting (titles), single panels refer to a given combination of the assumptions of worm-level regulation via density-dependence in egg production (“Worm-level”, columns) and snail-level regulation via explicit snail modelling (“Snail-level”, rows). Each line depicts results from a single model. The degree of regulation assumed at human level via anti-reinfection immunity (“Human-level”) is highlighted with different colours, from Absent to Strong, according to the legend above each figure. A single panel shows the simulated egg counts on the y-axis (mean epg is displayed, over age group and 100 stochastic realizations of the model) by age on the x-axis. The age-intensity profiles are displayed at stable pre-control settings, by varying the degree of regulating mechanism in humans (colours). Age bins are defined as to all be equally sized. (PDF) [file pntd.0012464.s003.pdf]

S1 Appendix

Age-intensity profiles

Age-intensity profiles for the complete set of modelling scenarios. For each choice of the age-exposure function and each endemicity setting (titles), single panels refer to a given combination of the assumptions of worm-level regulation via density-dependence in egg production (“Worm-level”, columns) and snail-level regulation via explicit snail modelling (“Snail-level”, rows). Each line depicts results from a single model. The degree of regulation assumed at human level via anti-reinfection immunity (“Human-level”) is highlighted with different colours, from Absent to Strong, according to the legend above each figure. A single panel shows the simulated egg counts on the y-axis (mean epg, over age group and 100 stochastic realizations of the model) by age on the x-axis. Age bins are defined as to all be equally sized.

1. Assumption for the age-exposure function: “Model-based”

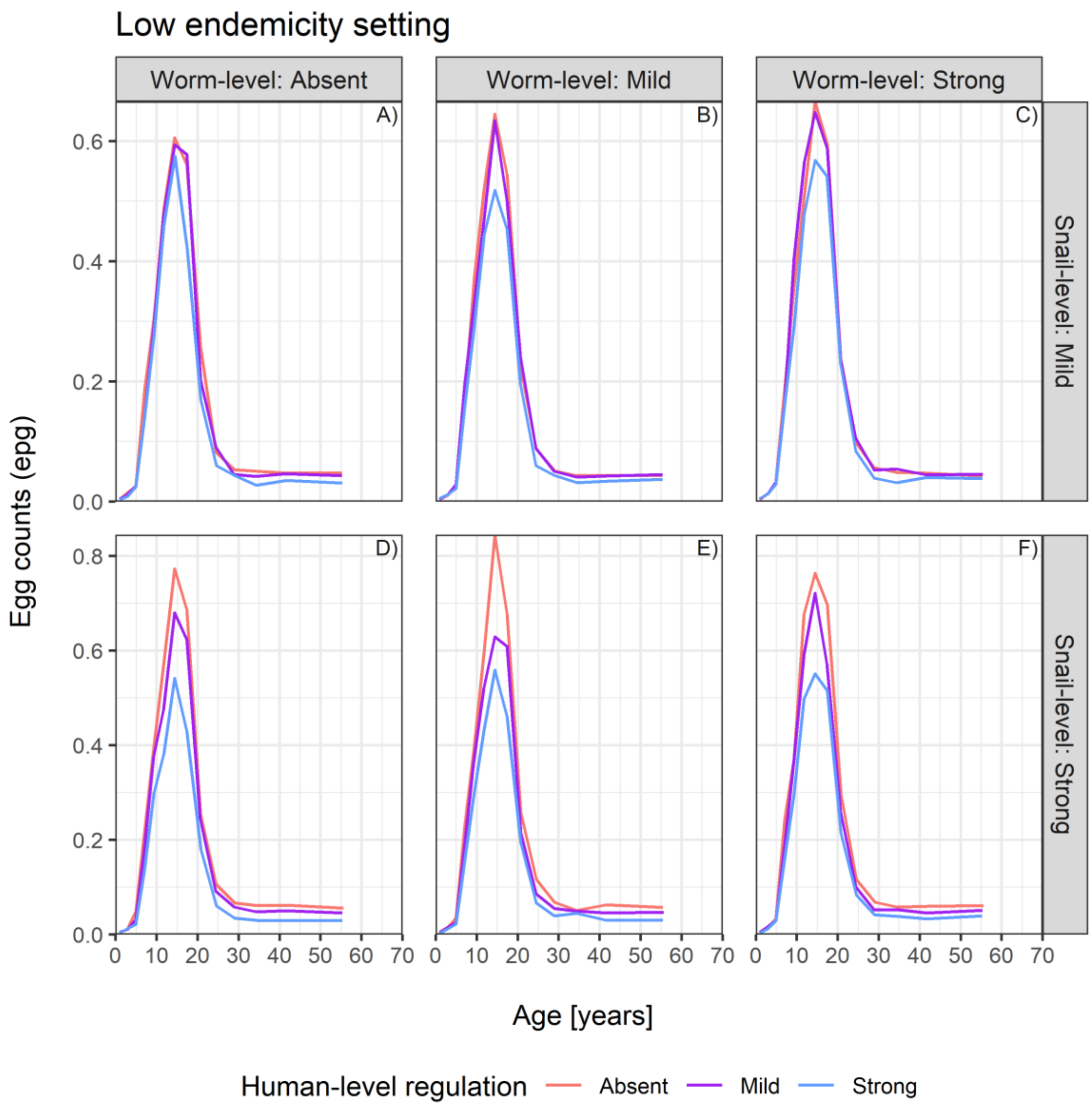

Low endemicity setting
Worm-level: Absent
Worm-level: Mild
Worm-level: Strong
Snail-level: Mild
Snail-level: Strong
A)
B)
C)
D)
E)
F)
Egg counts (epg)
Age [years]
Human-level regulation
Absent
Mild
Strong

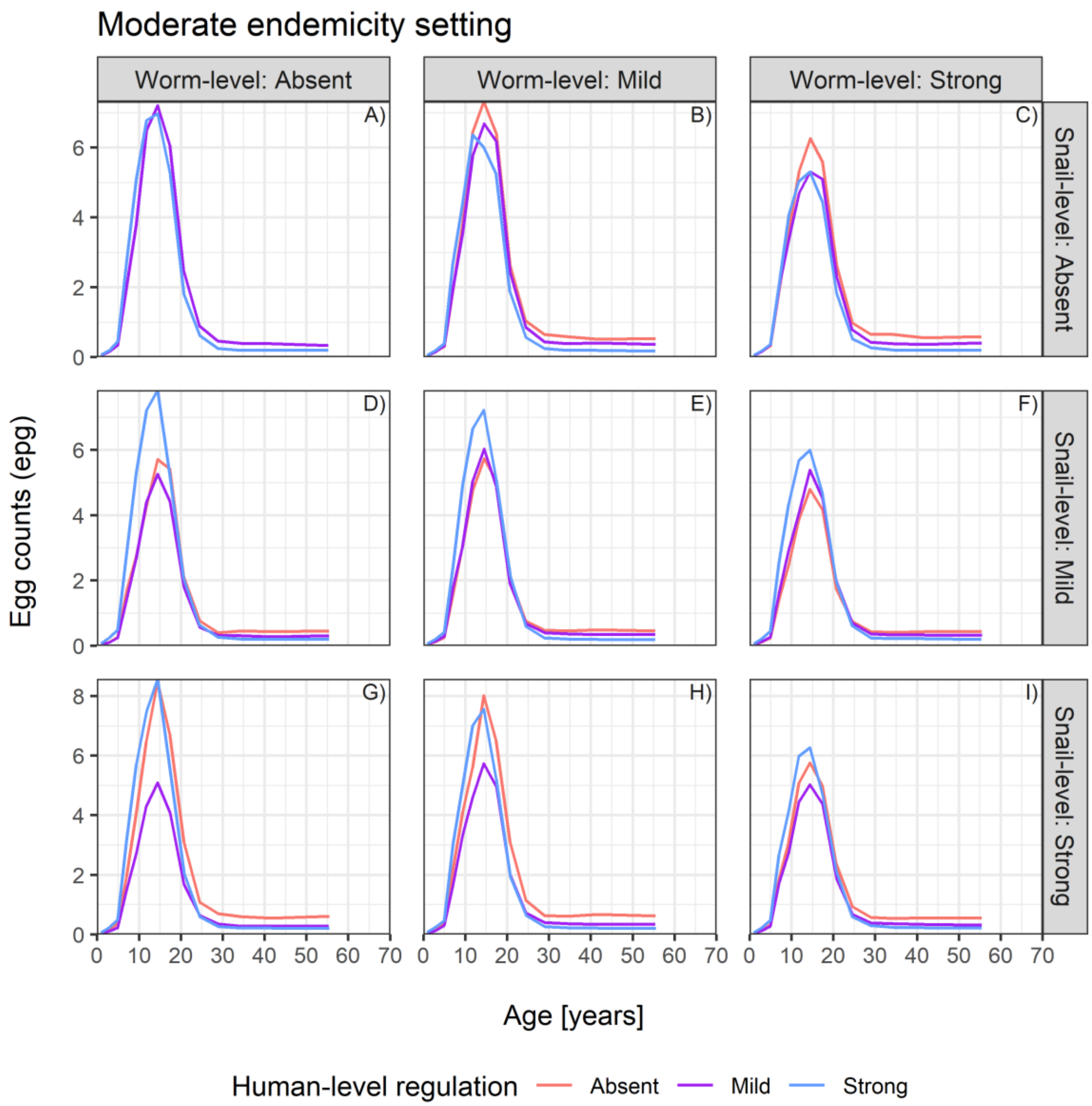
Moderate endemicity setting
Worm-level: Absent
Worm-level: Mild
Worm-level: Strong
Snail-level: Absent
Snail-level: Mild
Snail-level: Strong
A)
B)
C)
D)
E)
F)
G)
H)
I)
Egg counts (epg)
Age [years]
Human-level regulation
Absent
Mild
Strong

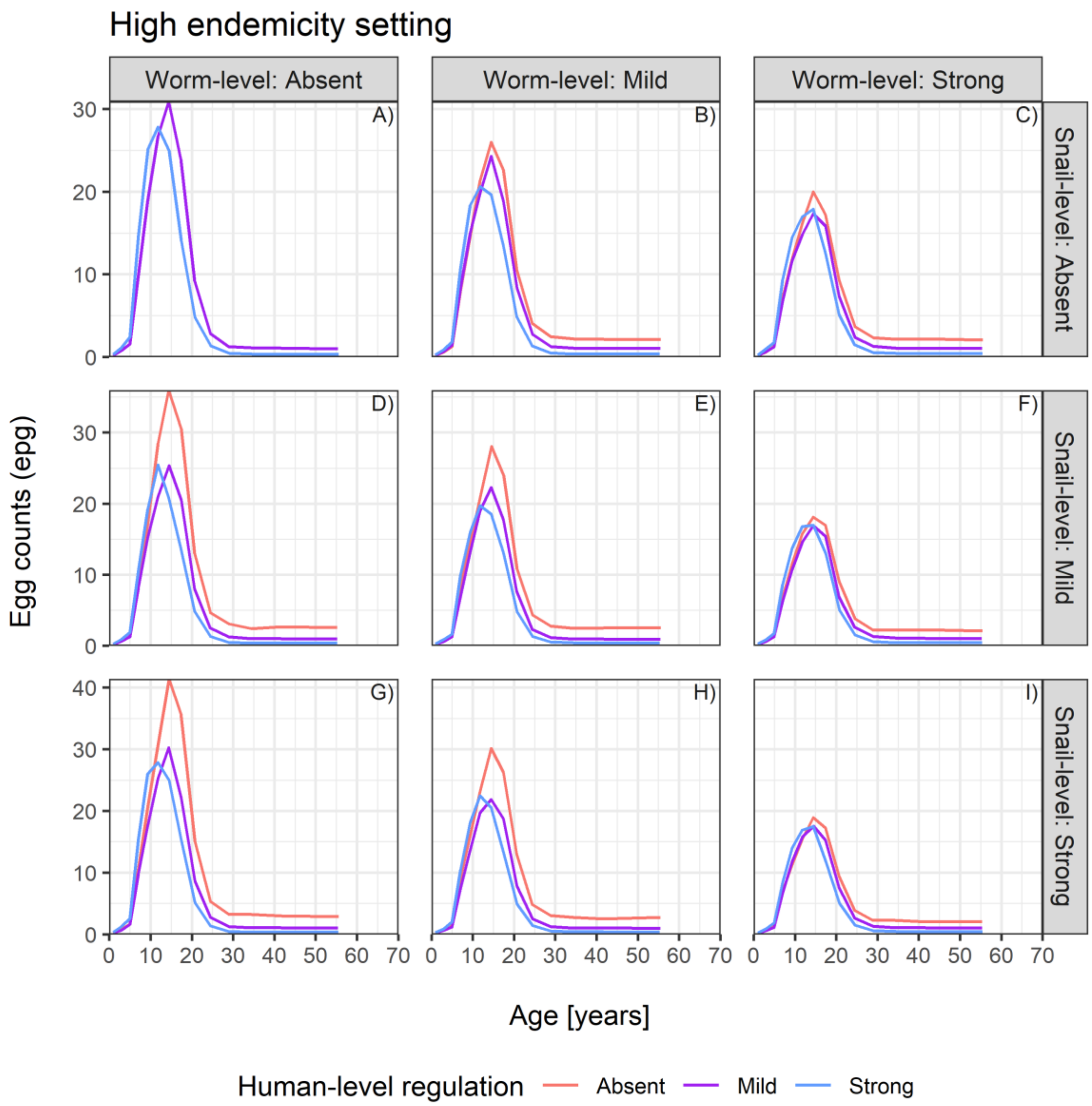
High endemicity setting
Worm-level: Absent
Worm-level: Mild
Worm-level: Strong
Snail-level: Absent
Snail-level: Mild
Snail-level: Strong
A)
B)
C)
D)
E)
F)
G)
H)
I)
Egg counts (epg)
Age [years]
0
10
20
30
40
50
60
70
Human-level regulation
Absent
Mild
Strong

2. Assumption for the age-exposure function: "Based on water contacts"

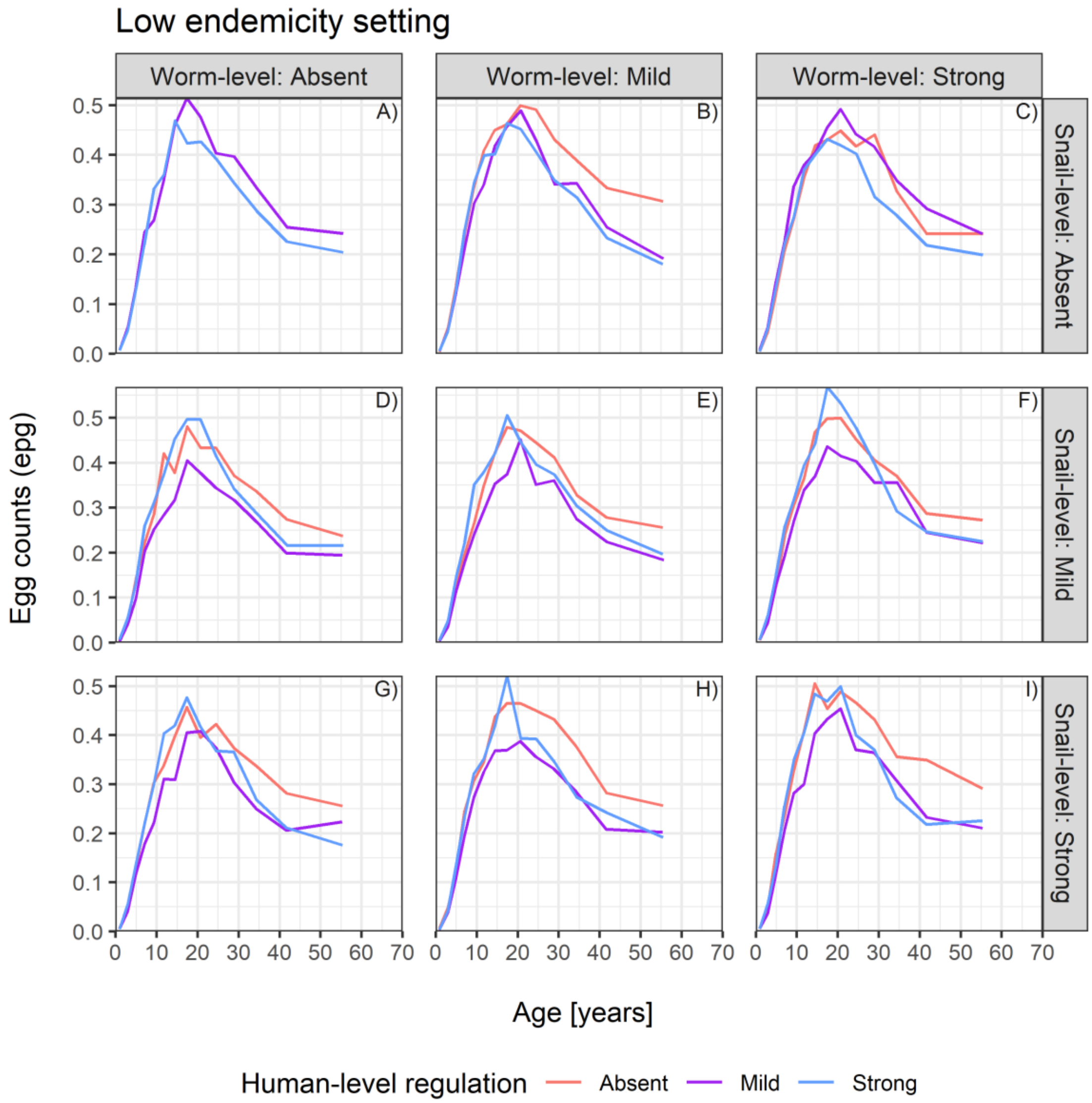

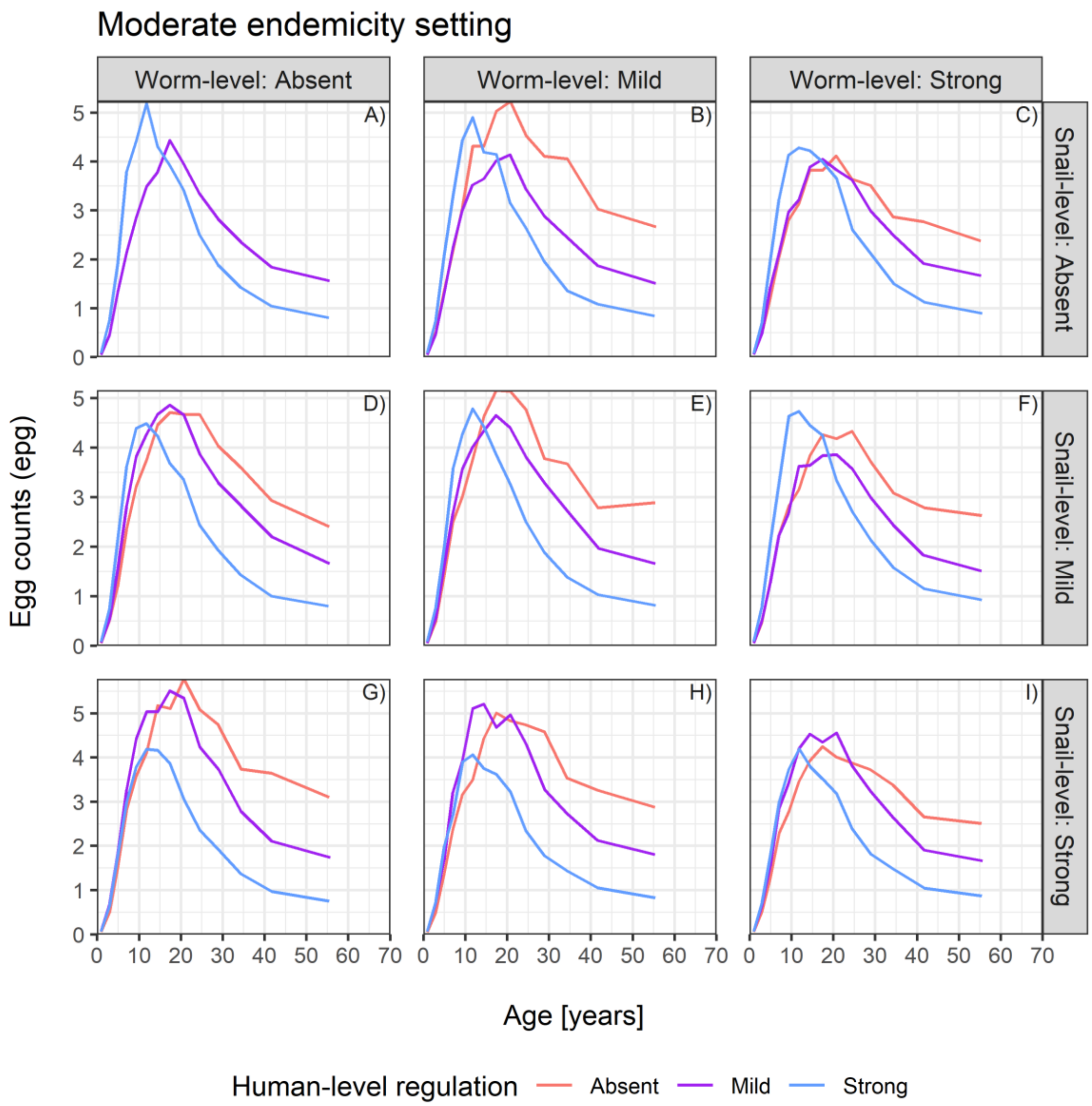

Moderate endemicity setting
Worm-level: Absent
Worm-level: Mild
Worm-level: Strong
Snail-level: Absent
Snail-level: Mild
Snail-level: Strong
A)
B)
C)
D)
E)
F)
G)
H)
I)
Egg counts (epg)
Age [years]
Human-level regulation
Absent
Mild
Strong

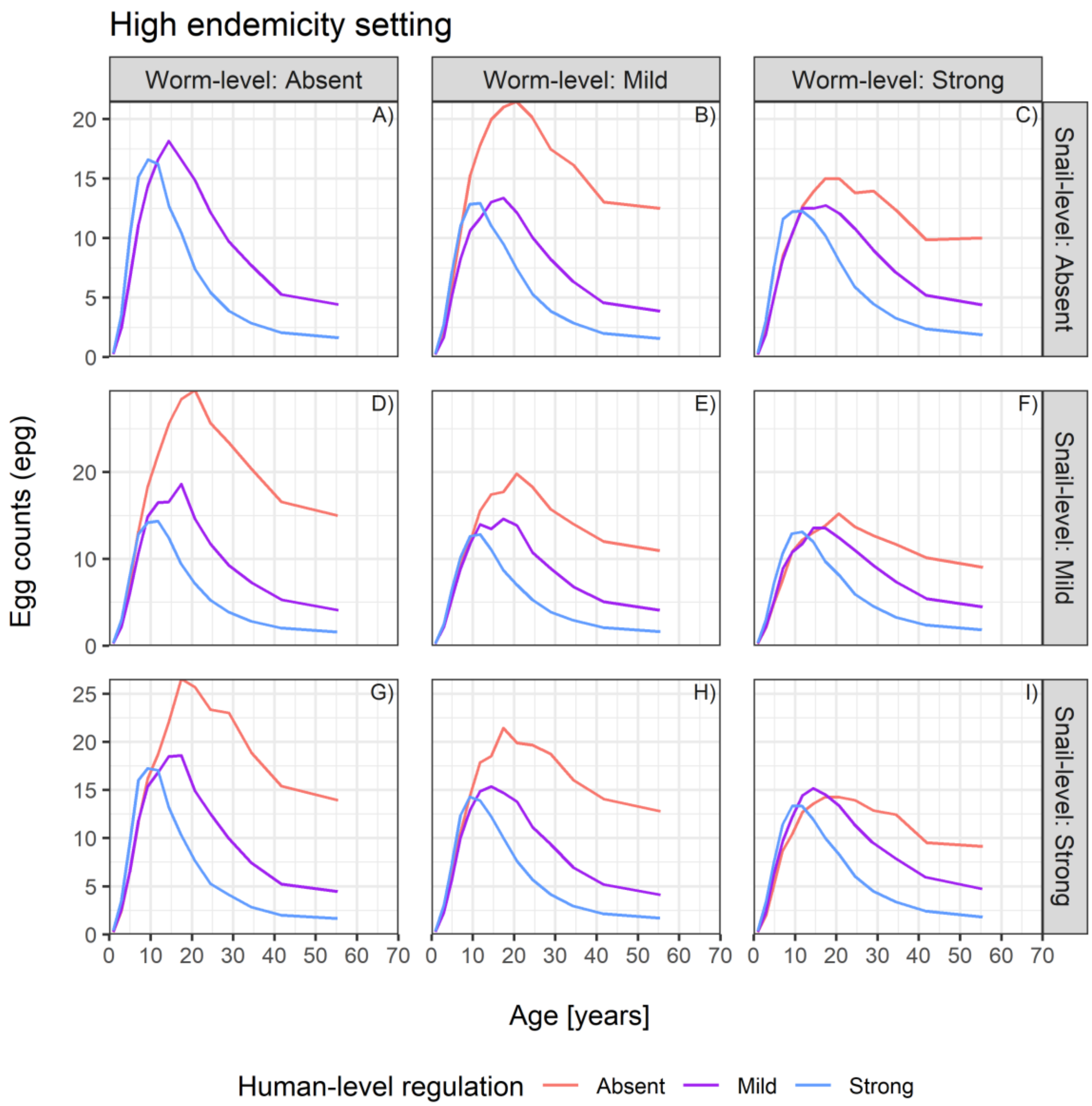
High endemicity setting
Worm-level: Absent
Worm-level: Mild
Worm-level: Strong
Snail-level: Absent
Snail-level: Mild
Snail-level: Strong
A)
B)
C)
D)
E)
F)
G)
H)
I)
Egg counts (epg)
Age [years]
Human-level regulation
Absent
Mild
Strong
