# Supplementary material for: Revisiting the impact of Schistosoma mansoni regulating mechanisms on transmission dynamics using SchiSTOP, a novel modelling framework
Source: PLoS Negl Trop Dis. 2024 Sep 20;18(9):e0012464. doi: 10.1371/journal.pntd.0012464 (PMC11414988; doi:10.1371/journal.pntd.0012464)

S2 Appendix

Effectiveness of treatment and prevalence bounce-back

Prevalence timelines in SAC during and after treatment. For each choice of the age-exposure function and endemicity setting (titles), single panels refer to a given combination of the assumptions of human-level regulation via anti-reinfection immunity ("Human-level", columns) and snail-level regulation via explicit snail modelling ("Snail-level", rows). The degree of regulation assumed at worm level density-dependence in egg production ("Worm-level") is highlighted with different colours, from Absent to Strong, according to the legend above each figure. A single panel shows the infection prevalence in school-aged children on the y-axis (mean of 100 stochastic realizations of the model, single runs as shaded lines) by round of treatment on the x-axis. For all scenarios, treatment is annually administered to 5-15 years old individuals with 10 repeated rounds, a coverage of 75% of the target population, 5% of target population systematically untreated, and a drug efficacy of 86%.

1. Assumption for the age-exposure function: "Model-based"

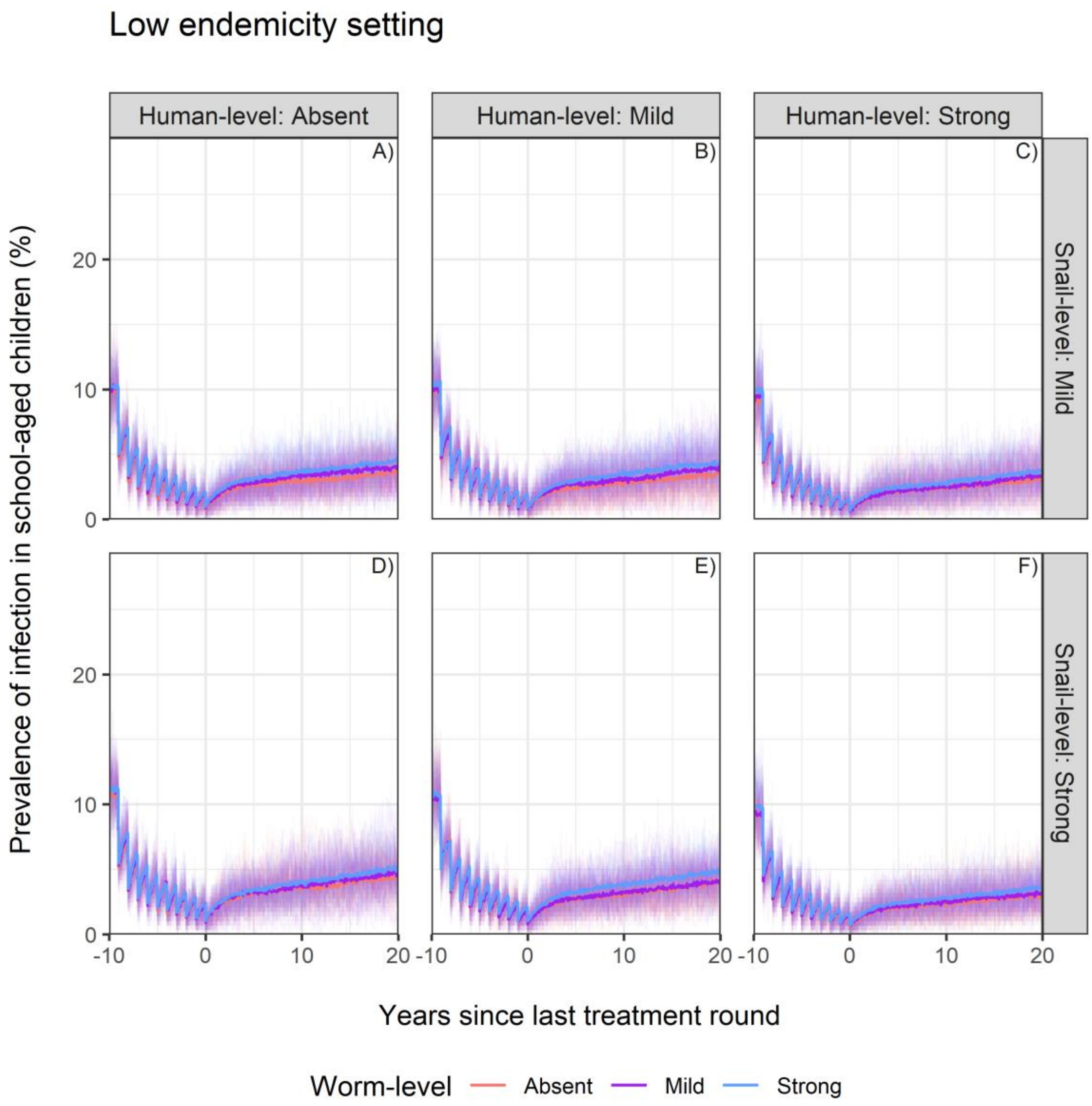

Low endemicity setting
Human-level: Absent
Human-level: Mild
Human-level: Strong
Snail-level: Mild
Snail-level: Strong
A)
B)
C)
D)
E)
F)
Prevalence of infection in school-aged children (%)
0
10
20
-10
0
10
20
Years since last treatment round
Worm-level
Absent
Mild
Strong

Moderate endemicity setting

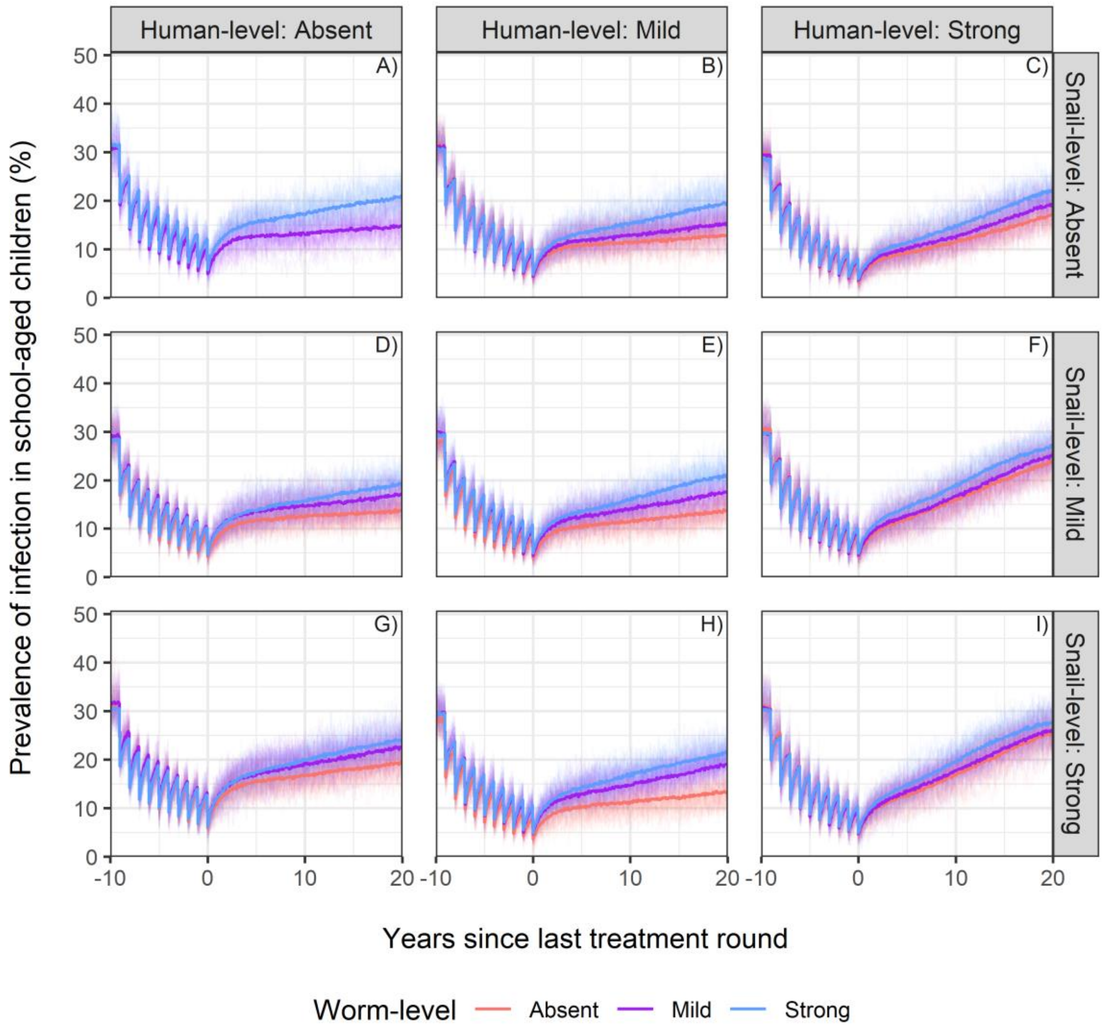

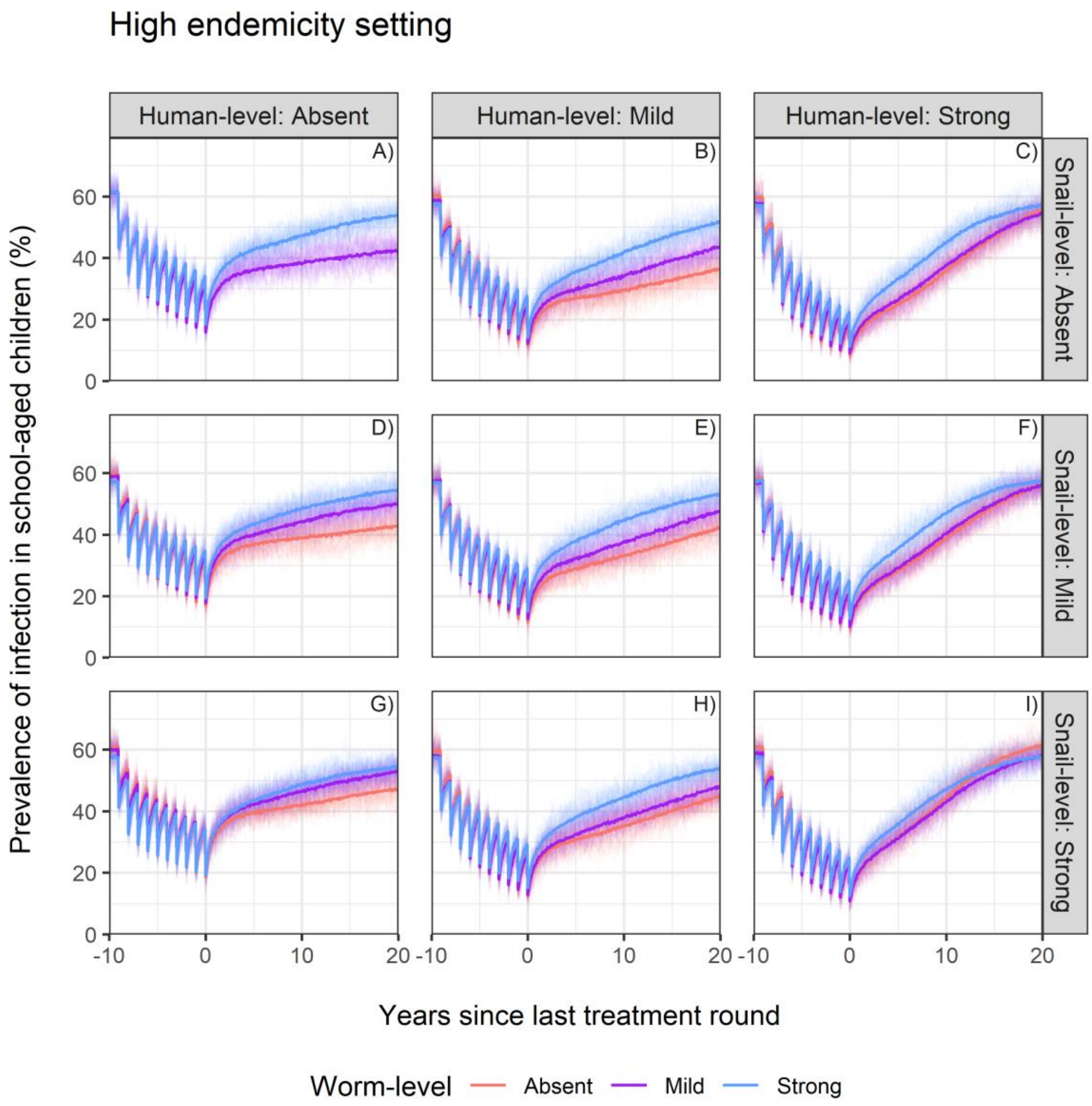

2. Assumption for the age-exposure function: "Based on water contacts"

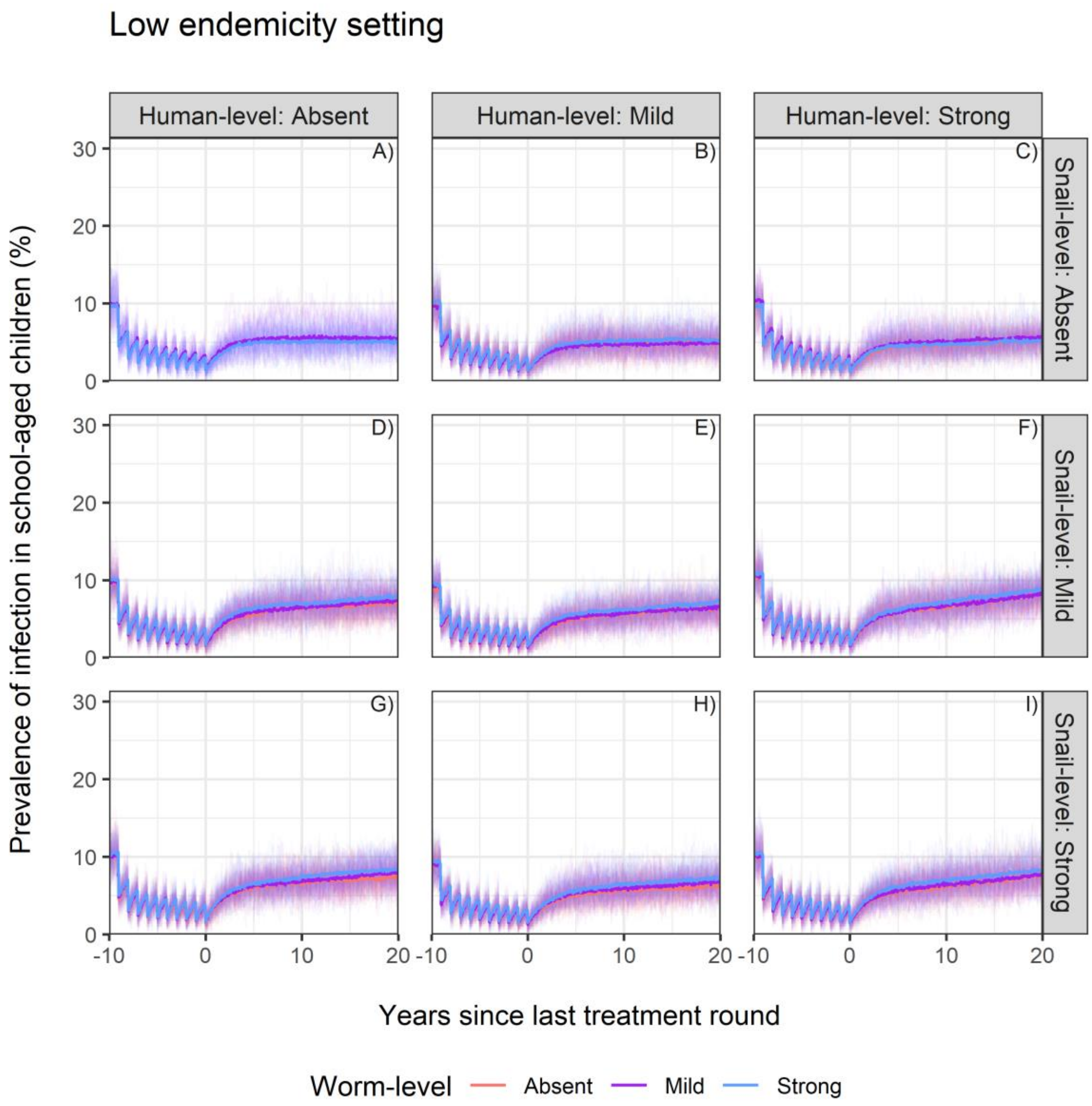

Low endemicity setting
Human-level: Absent
Human-level: Mild
Human-level: Strong
Snail-level: Absent
Snail-level: Mild
Snail-level: Strong
A)
B)
C)
D)
E)
F)
G)
H)
I)
Prevalence of infection in school-aged children (%)
0
10
20
30
-10
0
10
20
Years since last treatment round
Worm-level
Absent
Mild
Strong

Moderate endemicity setting

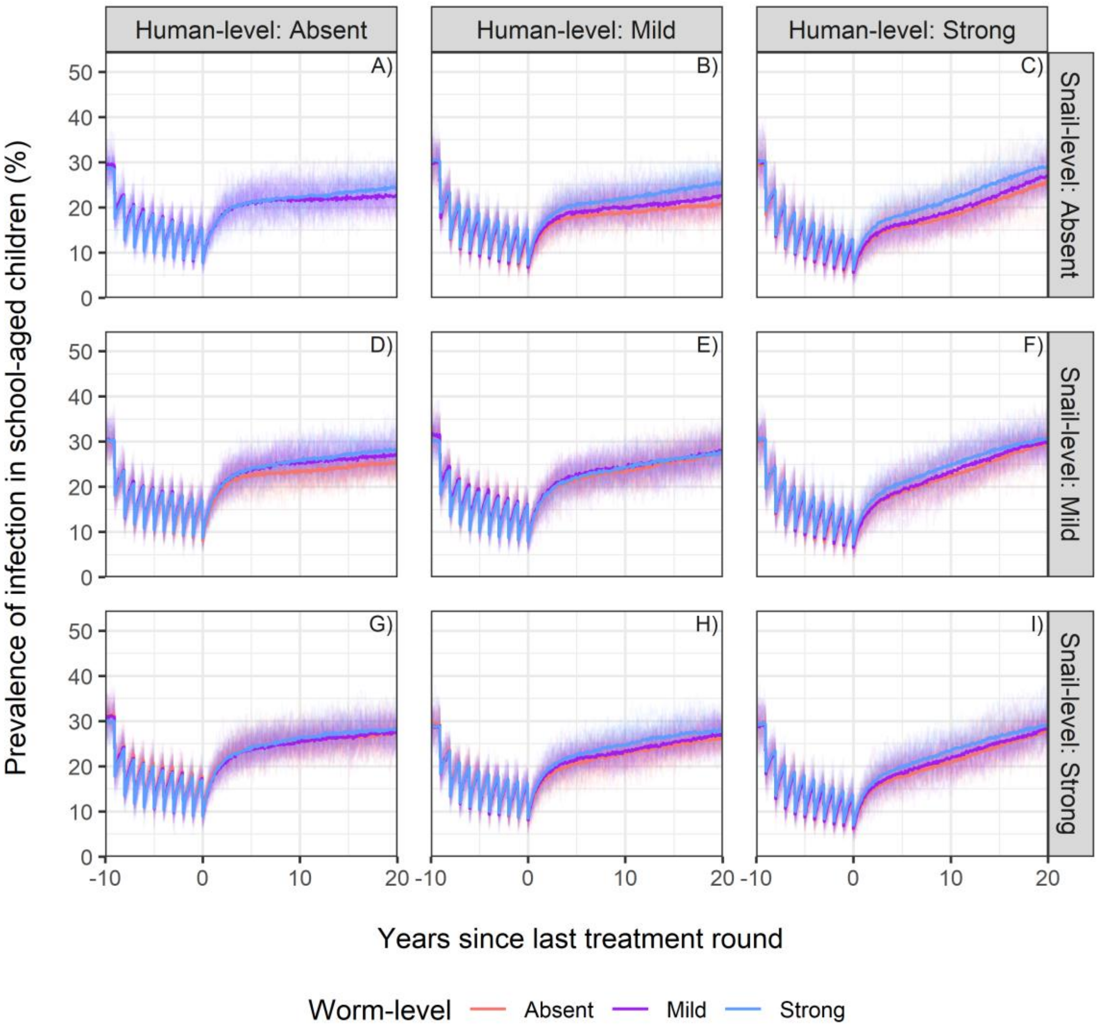

High endemicity setting

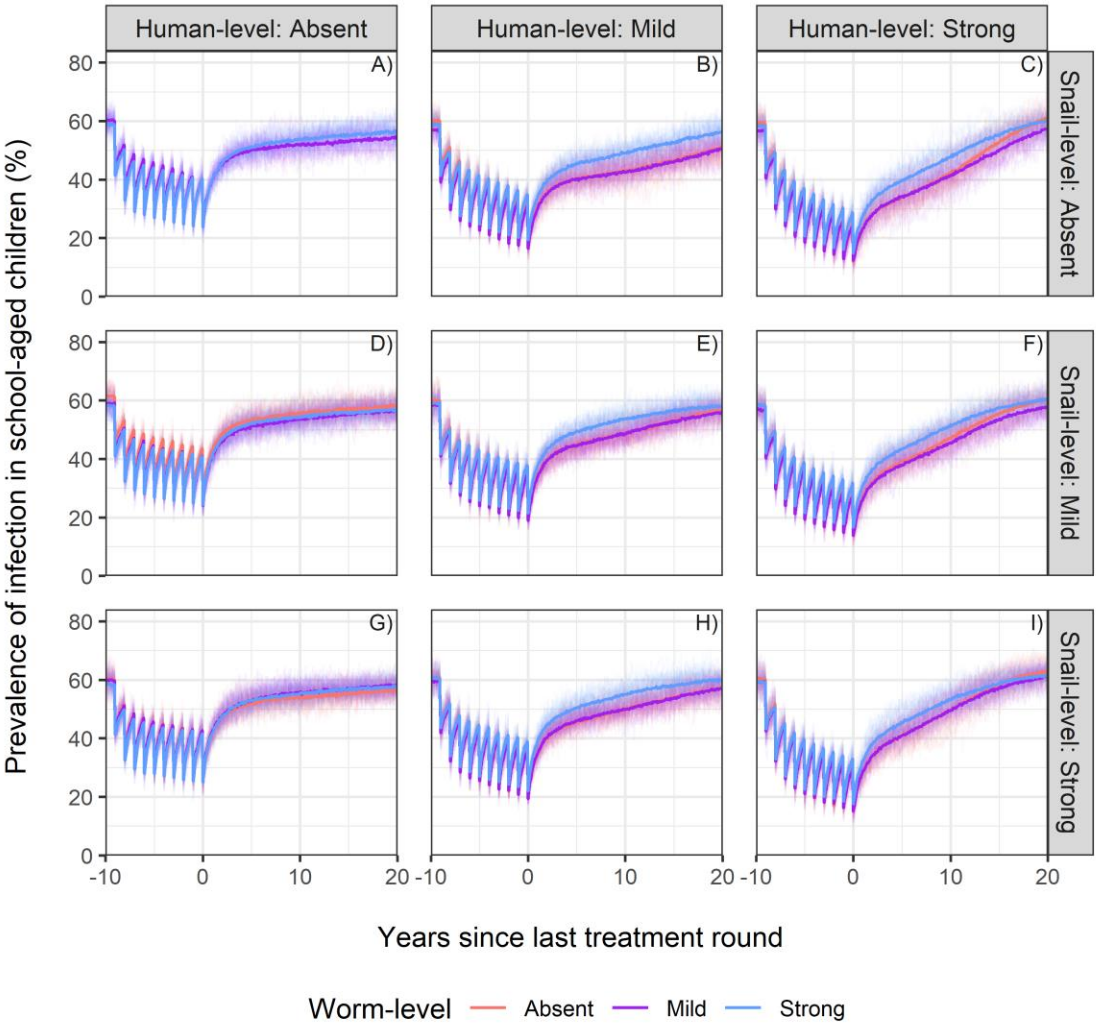

Supplement: S2 Appendix — For each choice of the age-exposure function and endemicity setting (titles), single panels refer to a fixed combination of the assumptions of human-level regulation via anti-reinfection immunity ("Human-level", columns) and snail-level regulation via explicit snail modelling ("Snail-level", rows). The degree of regulation assumed at worm level density-dependence in egg production ("Worm-level") is highlighted with different colours, from Absent to Strong, according to the legend above each figure. A single panel shows the infection prevalence in school-aged children on the y-axis (mean of 100 stochastic realizations of the model, single runs as shaded lines) by round of treatment on the x-axis. For all models, treatment is annually administered to 5–15 years old individuals with 10 repeated rounds, a coverage of 75% of the target population, 5% of target population systematically untreated, and a drug efficacy of 86%. (PDF) [file pntd.0012464.s004.pdf]
