# Supplementary material for: Revisiting the impact of Schistosoma mansoni regulating mechanisms on transmission dynamics using SchiSTOP, a novel modelling framework
Source: PLoS Negl Trop Dis. 2024 Sep 20;18(9):e0012464. doi: 10.1371/journal.pntd.0012464 (PMC11414988; doi:10.1371/journal.pntd.0012464)
Supplement: S3 Appendix — (PDF) [file pntd.0012464.s005.pdf]

S3 Appendix – Sensitivity analysis varying drug efficacy

We performed a sensitivity analysis of the drug efficacy (in the model defined as portion of adult worms killed by MDA with praziquantel), by varying the parameter between two values: 80% and 90%. The value of 86% used for the main analysis falls in this range.

For each choice of the parameter, we ran all the models as described in the "Simulations" section of the main text. We further computed the probability for each model to reach elimination as a public health problem (EPHP) and interruption of transmission.

Feasibility to reach the control targets

In line with the results described in the main text, successful models can still differ in their predictions of probability to reach the control targets, despite reproducing similar epidemiological patterns in terms of pre-control age-intensity profiles and bounce-back of prevalence after treatment. Results that follow include only such selected successful models (i.e. those that combined an age-exposure function based on water contacts with a mild or strong degree of regulation at both snail- and human- level), with the exception of the model based on only strong worm-level regulation and model-based exposure function, as current models for policy use the same assumptions.

Reaching control targets with 10-year annual MDA to school-aged children

We found that all successful models agree that treating SAC only with 10 years of annual MDA will not allow to reach the EPHP in high or moderate endemicity settings, irrespective of the value set for the drug efficacy. In low endemicities, we start from a setting where the prevalence of high intensity infection in SAC is already below 10% (EPHP has already been met). Here, the target of interest is the IOT and none of the models predict a positive probability to reach this target despite 10 rounds of annual MDA to SAC. Results are different for the model assuming only strong worm-level regulation and the model-based exposure function, used here as reference for common assumptions. In fact, this model predicts a probability of 76% to reach EPHP in moderate endemicity setting, if drug efficacy is 90%.

The impact of community-wide annual MDA

The figures below report the predictions to the control targets assuming a strategy of community-wide annual MDA, by varying the parameter of drug efficacy. The main effect of varying the drug efficacy is a decline or an improvement of the MDA effectiveness. This is reflected in the absolute predictions which can differ from the figures in the main text. However, the trend across models with varying regulating assumptions is not impacted, supporting the main message of this study.

Worm killing rate = 0.8

Probability to elimination as a public health problem

Low endemicity

Human-level	Worm-level: Absent	Mild	Strong	Snail-level
Mild	100	100	100	Mild
Mild	100	100	100	Strong
Strong	100	100	100	Mild
Strong	100	100	100	Strong

Moderate endemicity

Human-level	Worm-level: Absent	Mild	Strong	Snail-level
Absent			100*	Absent
Mild	100	100	100	Mild
Mild	100	100	98	Strong
Strong	74	59	15	Mild
Strong	92	83	57	Strong

Probability
100
80
60
40
20
0

High endemicity

Human-level	Worm-level: Absent	Mild	Strong	Snail-level
Absent			91*	Absent
Mild	82	74	2	Mild
Mild	74	59	0	Strong
Strong	0	0	0	Mild
Strong	0	0	0	Strong

Worm-level

Worm killing rate = 0.9

Probability to elimination as a public health problem

Low endemicity

Human-level	Worm-level: Absent	Mild	Strong	Snail-level
Mild	100	100	100	Mild
Mild	100	100	100	Strong
Strong	100	100	100	Mild
Strong	100	100	100	Strong

Moderate endemicity

Human-level	Worm-level: Absent	Mild	Strong	Snail-level
Absent			100*	Absent
Mild	100	100	100	Mild
Mild	100	100	96	Strong

Probability
100
80
60

Human-level	Absent	Mild	Strong	Snail-level
Strong	82	79	35	Mild
Strong	95	95	67	Strong

Probability
40
20
0

High endemicity

Human-level	Absent	Mild	Strong	Snail-level
Absent			99*	Absent
Mild	96	91	24	Mild
Mild	91	82	6	Strong
Strong	0	0	0	Mild
Strong	0	0	0	Strong

Worm-level

Probability to reach elimination as a public health problem with a 10-year annual MDA to all individuals older than 2. The figure reports predictions for the probability (portion of the 100 stochastic runs monitored at 20 years after end of treatment) of reaching elimination as a public health problem (EPHP, prevalence of heavy infections in school-aged children < 10%), across models successful in reproducing all observed patterns for schistosomiasis. Successful models assume an age-exposure function based on water contacts. Regulating mechanisms can be at human-level ("Human-level", left side, via anti-reinfection immunity), snail-level ("Snail-level", right side, via density-dependence in population growth), and worm-level ("Worm-level", bottom, via density-dependence in egg production). *This model assumes a strong worm-level regulation only and the model-based age-exposure function. It was not successful in reproducing observed patterns within our modelling framework, but it is added here for reference as current models for policy use the same assumptions. For all models, treatment is annually administered to 2+ years old individuals with a 75% coverage of the target population, 5% of target population systematically untreated, and a drug efficacy of 80% and 90% in the top and bottom figure, respectively.

Worm killing rate = 0.8

Probability to interruption of transmission

Low endemicity

**Human-level**	Absent	Mild	Strong	**Snail-level**
Mild	57	51	50	Mild
Mild	62	55	45	Strong
Strong	34	32	27	Mild
Strong	45	31	35	Strong

Moderate endemicity

Human-level				Snail-level
Absent			1*	Absent
Mild	0	0	0	Mild
Mild	0	0	0	Strong
Strong	0	0	0	Mild

**Probability**
100
80
60
40

Strong	0	0	0	Strong	20
	Absent	Mild	Strong		0

High endemicity

Absent			0*	Absent
Mild	0	0	0	Mild
Mild	0	0	0	Strong
Strong	0	0	0	Mild
Strong	0	0	0	Strong
	Absent	Mild	Strong	

Worm-level

Worm killing rate = 0.9

Probability to interruption of transmission

**Human-level**	Low endemicity			**Snail-level**
Mild	64	59	65	Mild
Mild	64	53	56	Strong
Strong	53	47	47	Mild
Strong	55	48	43	Strong
	Absent	Mild	Strong	

Moderate endemicity

					**Probability**
Absent			2*	Absent	100
Mild	0	0	1	Mild	80
Mild	0	0	0	Strong	60
Strong	0	0	0	Mild	40
Strong	0	0	0	Strong	20
	Absent	Mild	Strong		0

High endemicity

Absent			0*	Absent
Mild	0	0	0	Mild
Mild	0	0	0	Strong
Strong	0	0	0	Mild
Strong	0	0	0	Strong
	Absent	Mild	Strong	

Worm-level

Fig 5. Probability to reach interruption of transmission by 10-year annual MDA to all individuals older than 2. The figure reports predictions for the probability (portion of the 100 stochastic runs

monitored at 20 years after end of treatment) of reaching interruption of transmission (0% prevalence of any infection in school-aged children), across models successful in reproducing all observed patterns for schistosomiasis. Successful models assume an age-exposure function based on water contacts. Regulating mechanisms can be at human-level (“Human-level”, left side, via anti-reinfection immunity), snail-level (“Snail-level”, right side, via density-dependence in population growth), and worm-level (“Worm-level”, bottom, via density-dependence in egg production). *This model assumes a strong worm-level regulation only and the model-based age-exposure function. It was not successful in reproducing observed patterns within our modelling framework, but it is added here for reference as current models for policy use the same assumptions. For all models, treatment is annually administered to 2+ years old individuals with a 75% coverage of the target population, 5% of target population systematically untreated, and a drug efficacy of 80% and 90% in the top and bottom figure, respectively.
